# Preparation of Thioaminals in Water

**DOI:** 10.3390/molecules27051673

**Published:** 2022-03-03

**Authors:** Lídia A. S. Cavaca, Rafael F. A. Gomes, Carlos A. M. Afonso

**Affiliations:** Research Institute for Medicines (iMed.ULisboa), Faculty of Pharmacy, University of Lisbon, Av. Prof. Gama Pinto, 1649-003 Lisboa, Portugal; l.cavaca@campus.fct.unl.pt

**Keywords:** sustainability, aldehyde, catalysis

## Abstract

The presence of sulfur–carbon bonds is transversal to several areas of chemistry, e.g., drug discovery, materials, and chemical biology. However, a lack of efficient and sustainable procedures for the preparation of thioaminals, the *N,S*-analogues of *O,O*-acetals, contributes to this functional group often being overlooked by the scientific community. In this work is described the formation of thioaminals in water promoted by copper(II) triflate.

## 1. Introduction

The preparation of sulfur-rich frameworks has been a relevant topic for organic chemists, given its importance in materials [1,2,3,4] and medicinal chemistry [5,6]. The need for the formation of C-S bonds led to developments such as the click thio-ene reaction, a cornerstone of material chemistry [1]. Additionally, thio-Michael additions are often used for the derivatization of activated olefins, a strategy widely used for the functionalization of materials and peptides [6,7].

**Figure 1:**
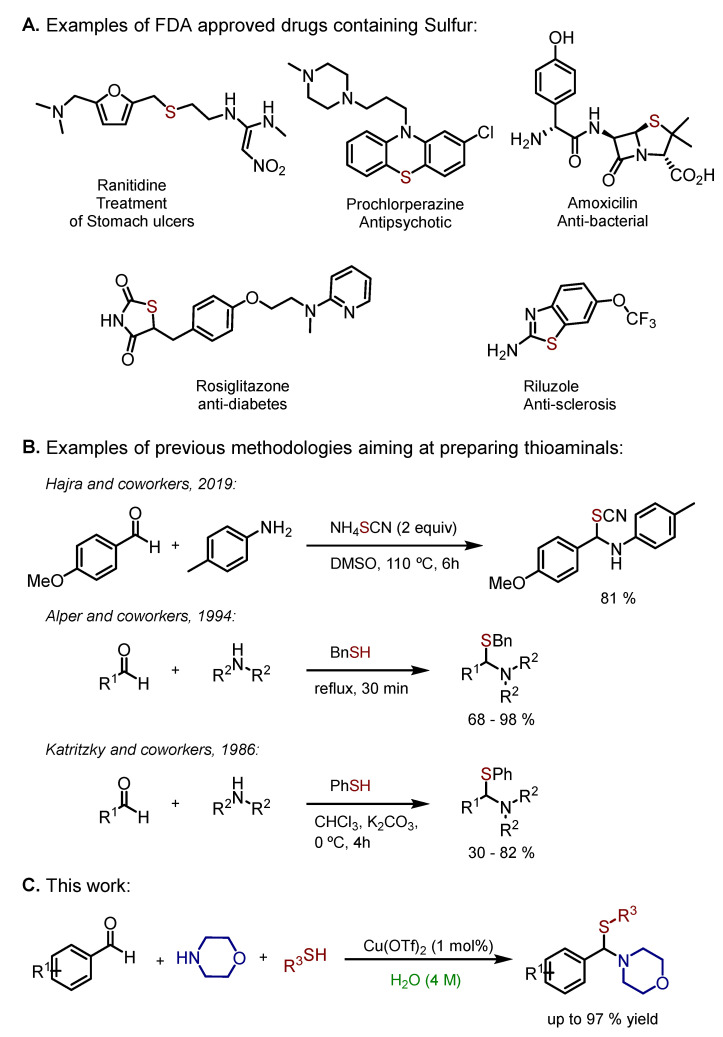
Importance of thiol in drug development (**A**); selected examples for the preparation of thioaminals (**B**); [20,21,22,23] this work (**C**).

Focusing on the medicinal chemistry area, sulfur is a key constituent of over 20% of FDA-approved drugs [8,9], being responsible for favorable interactions on the targeted active site (Figure 1A) [10]. Amongst the sulfur-containing scaffolds, heterocycles such as thiazolidines and thiazolidinones have revealed a variety of biological activities [11,12,13], leading to abundant procedures for the formation of these cyclic *S*,*N*-aminals (thioaminals) [14,15,16,17,18,19]. However, reports on the formation of acyclic thioaminals are scarce, often requiring harsh conditions including hazardous reagents, affording poor yields or being limited to non-isolated intermediaries (Figure 1B) [20,21,22,23].

Recently, there has been an increased concern about the development of sustainable methodologies [24,25,26,27], including procedures in water [28,29,30,31,32]. With this in mind, our group (i) focused on the valorization of raw renewable materials such as furfural and hydroxymethylfurfural (HMF) [33,34,35] and (ii) reported on the use of water as a green reaction media for the production of cyclopentenones [36] and, more recently, aminals from the condensation of aryl aldehydes with amines [37]. This addresses one of the 12 principles of green chemistry [24], namely the use of less hazardous/toxic chemicals. The structural resemblance of aminals and thioaminals led us to enquire if a similar methodology could be applied to their formation. Herein is described the formation of thioaminals under aqueous conditions promoted by copper(II) triflate (Figure 1C).

## 2. Results

Selecting furfural, morpholine and thiophenol as model substrates, a screening of conditions was initially performed to identify the most suitable catalyst and solvent (Table 1). The formation of thioaminal **1** in the presence of catalytic copper(II) triflate (1 mol%) in aqueous media afforded the desired product in 84% isolated yield after 10 min of reaction (Table 1, entry 3). In the absence of catalyst, 100% of starting furfural was recovered (Table 1, entry 1), while an increase in catalyst loading to 10 mol% resulted in full conversion to the corresponding cyclopentenone (Table 1, entry 2) [36,38]. Other attempted metal-based catalysts (i.e., CuSO_4_, FeCl_3_, AlCl_3_ and ZnCl_2_) and Brønsted acids (i.e., triflic acid) also led to the formation of cyclopentenone as a side-product (Table 1, entries 7–11) [36,38]. Performing the reaction under neat conditions or by changing the solvent to ethanol or acetonitrile did not allow full conversion and caused a visible contamination with cyclopentenone (Table 1, entries 4–6). Thus, water was considered the most suitable solvent for this reaction.

**Scheme 1:**
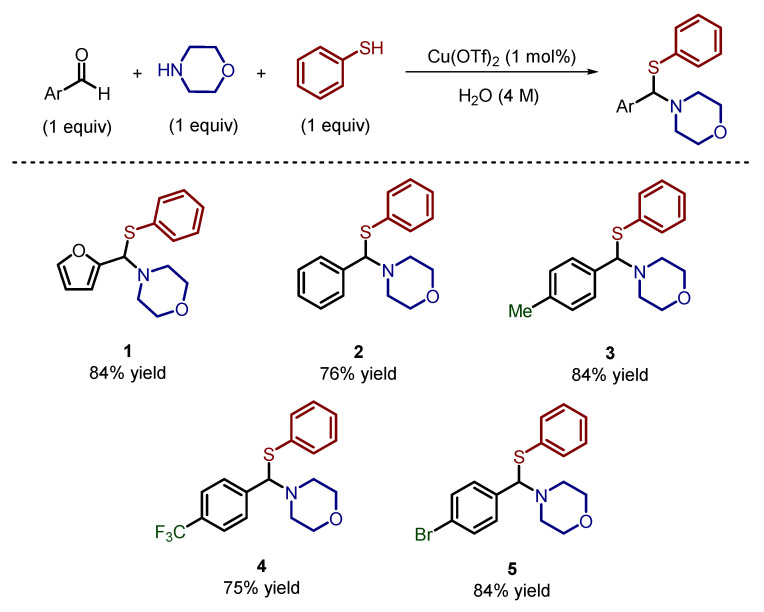
Scope of aryl aldehydes for the formation of thioaminals.

Following the best selected conditions, we expanded the scope to a plethora of aryl and alkyl aldehydes (Scheme 1). First, we reacted benzaldehyde, morpholine and thiophenol to prepare thioaminal **2** in 76% yield. Electron-rich aromatic aldehydes, such as 4-NMe_2_-benzaldehyde and 4-OMe-benzaldehyde, resulted in no reaction or incomplete reaction (65% conversion), and were impossible to isolate due to the product instability. Similar results were obtained when alkyl aldehydes were employed, resulting in a complex mixture.

Counterintuitively, 4-Me-benzaldehyde afforded a better yield than 4-CF_3_-benzaldehyde, and a similar yield was obtained for the 4-Br-benzaldehyde derivative. Being electron poor, the formation of **4** and **5** should be favored since the aldehyde is more prone to undergo condensation with the secondary amine. Moreover, the use of 4-NO_2_-benzaldehyde only gave a 50% conversion to the corresponding thioaminal, without the possibility of purification. These unexpected results led us to conclude that other factors such as the stability of the constructs may play a role when analyzing the success of these reactions.

To our surprise, we did not observe the formation of the *N*,*N* aminal product in this aldehyde scope, which clearly indicates that the *N*,*S* thioaminals are thermodynamically more favorable. However, if we allowed for the reaction to proceed for longer periods of time, the yields would drop significantly. In this situation, no aldehyde is observed as well, indicating another decomposition pathway than the simple hydrolysis of the thioaminal. Focusing on isolation, we stumbled upon a major challenge, which was the hydrolysis of the thioaminals. Simple operations such as extraction, even at higher pH using NaHCO_3_ (sat aq.), led to hydrolysis and subsequently low yields of the products. Freeze-drying the solution, followed by washing the corresponding powders with cold water, significantly increased the isolated yields. Contrary to other prepared thioaminals, product **1,** obtained from furfural, exhibited increased stability even during the work-up extraction with MTBE.

**Scheme 2:**
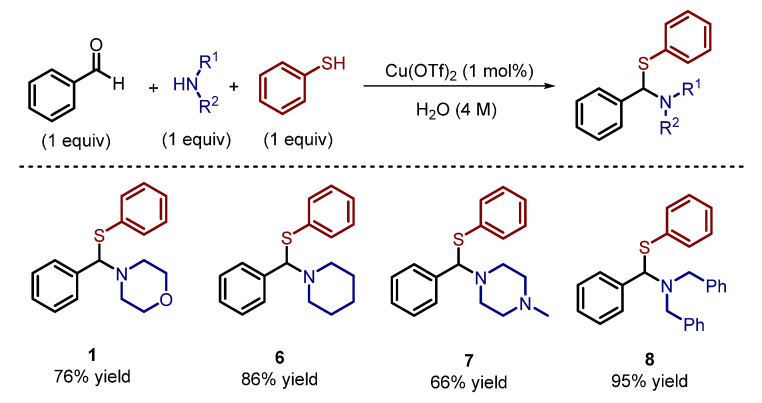
Scope of alkyl amines for the formation of thioaminals.

A search for secondary aliphatic and aromatic amines was also performed (Scheme 2). In addition to morpholine, other aliphatic amines such as piperidine, *N*-methyl piperazine and dibenzyl amine were tolerated under the reaction conditions, affording the corresponding thioaminals **6**–**8** in a good to excellent yield. These compounds exhibited hygroscopic properties, resulting in fast hydrolysis at room temperature or at 5 °C. Bulky aliphatic amines, such as di-isopropylamine, did not react and the reagents were fully recovered. Surprisingly, diethylamine was also not a successful amine. An aromatic amine, *N*-methyl aniline, was employed under the reaction conditions, but no reaction occurred.

**Scheme 3:**
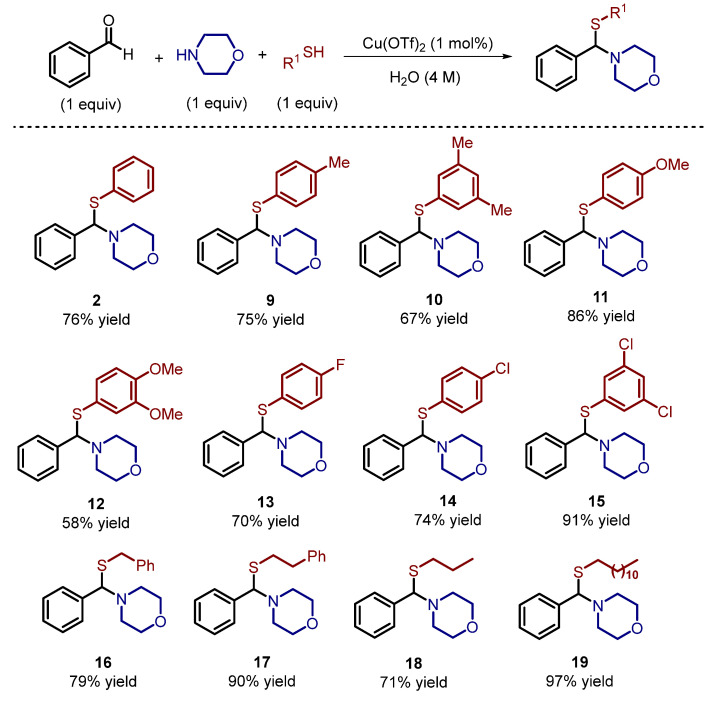
Scope of aryl and alkyl thiols for the formation of thioaminals.

Next, a series of aryl and aliphatic thiols were reacted with benzaldehyde and morpholine (Scheme 3). The reaction withstood electron-rich thiols such as 4-Me, 3,5-diMe, 4-OMe and 3,4-diOMe thiophenol yielding the thioaminal products **9**–**12** in good yield. Electron-poor thiol such as 4-F, 4-Cl and 3,5-diCl thiophenol also afforded the desired products, **13**–**15,** in good to excellent yields. A reaction with 4-NO_2_-thiophenol was also attempted, but only 63% conversion to the thioaminal was observed, without the possibility of purification. Finally, alkyl thiols also afforded products **16**–**19** in good yield, even when long-alkyl-chain thiols (i.e., dodecanethiol) were employed.

An additional reaction using three solid substrates (i.e., 4-Br-benzaldehyde, 4-Cl-thiophenol and *N*-acetylated piperazine) was performed in water. The corresponding thiominal was formed to some extent (68% conversion), indicating that solid substrates are more challenging and that the use of organic solvents might be necessary.

**Scheme 4:**
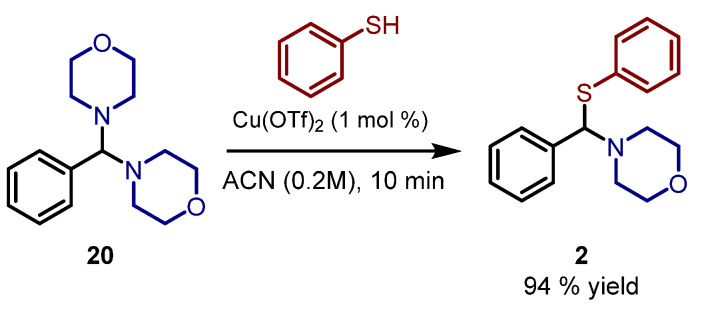
Amine/thiol exchange of aminals.

Intrigued by the selective formation of the thioaminal, a solution of the aminal in acetonitrile was reacted with thiophenol in the presence of a catalyst and a full conversion of aminal **20** was observed to the corresponding thioaminal **2**, isolated in 94% yield (Scheme 4).

**Figure 2:**
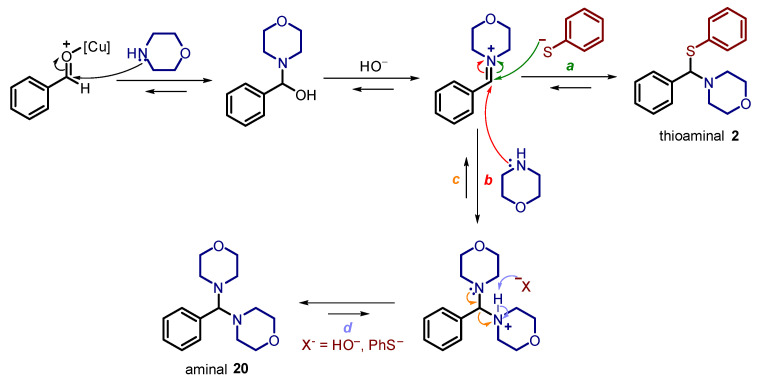
Proposed mechanism for the formation of thioaminals.

The proposed mechanism for the formation of the thioaminals depicted in Figure 2 is initiated by the formation of an iminium ion, which may either undergo further condensation with an amine (path a) or with a thiol (path b) to form the desired product. The fact that aminals in the presence of thiol lead to the formation of thioaminals suggests that even when the aminal is formed, its reversibility by the elimination of an amine generates the parent iminium ion, which will react with the thiol to form the product thioaminal.

**Figure 3:**
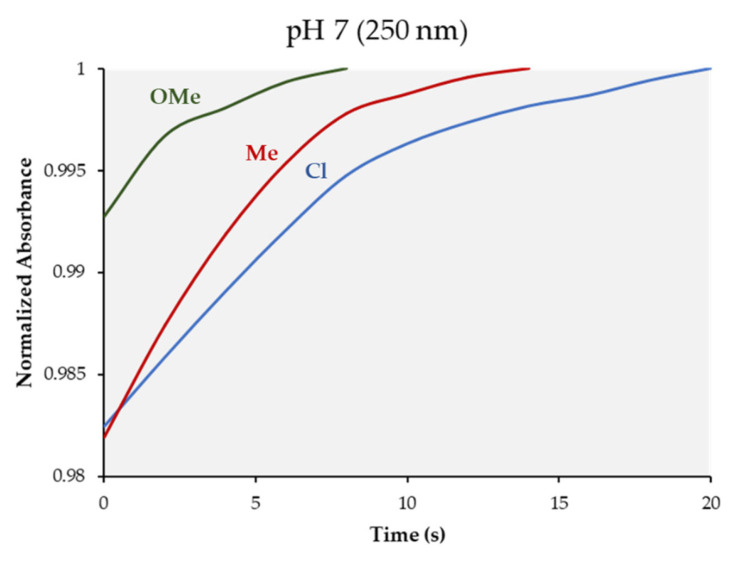
Hydrolysis evaluation by UV-vis spectroscopy of thioaminals **9**, **11** and **14**.

Following our previous studies on the stability of *N,N*-aminals, in which was observed that the electron-donating groups in the aldehyde stabilized the products, we enquired into the role of substituents in the arylthiol. To this end, we accessed the rates of hydrolysis of three thioaminals bearing electron-withdrawing (**14**) and electron-donating (**9,11**) *para* substituents by UV-vis spectroscopy, as depicted in Figure 3. We observed a fast hydrolysis of <20 s in these diluted conditions (see Supplementary Materials, Figures S21 and S23). Despite this, a trend was observed where electron-withdrawing substituents on the thiol slightly hindered the hydrolysis of the products.

## 3. Materials and Methods

### 3.1. General Information

All solvents were of analytical grade and distilled prior to use. All reagents were used as received from commercial suppliers. NMR spectra were recorded in a Bruker Fourier 300 or Bruker Advance 400 spectrometer. High-Resolution Mass Spectrometry (HRMS) results were recorded in a Thermo Scientific Q Exactive hybrid quadrupole-Orbitrap mass spectrometer (Thermo Scientific^TM^ Q Exactive^TM^ Plus, Waltham, Massachusetts, U.S.). ^1^H NMR, ^13^C NMR, HRMS and melting-point data are reported for all new compounds. The characterizations of reported compounds are in accordance with the literature. The UV stability measurements were traced in a Thermo Scientific^TM^ Evolution^TM^ 201 UV-visible spectrophotometer (Thermo Scientific, Waltham, Massachusetts, U.S.).

### 3.2. General Procedure for Cu(II)-Catalyzed Preparation of Thioaminals in Water

Aldehyde (0.942 mmol, 1 equiv) and morpholine (0.942 mmol, 1 equiv) were placed in a round-bottom flask followed by addition of aqueous stock solution of Cu(OTf)_2_ (1 mol%, 227 μL from 15 mg/mL stock solution). Then, thiol (0.942 mmol, 1 equiv) was added. The reaction was stirred at room temperature for 10 min and water removed by lyophilization to obtain pure thioaminals. Additional purification might have been required by washing with distilled water (pH 9–10). In the case of thioaminal **1**, an extraction with MTBE was performed.

### 3.3. Characterization Data for Thioaminals

*4-(furan-2-yl(phenylthio)methyl)morpholine* (**1**). Prepared according to general procedure using furfural (0.0905 g, 0.942 mmol, 1 equiv), morpholine (0.082 g, 0.942 mmol, 1 equiv) and thiophenol (0.1038 g, 0.942 mmol, 1 equiv). The compound was obtained as a yellow solid (0.2179 g, 84% yield) after extraction with MTBE (3 mL). ^1^H NMR (400 MHz, Acetone-*d_6_*): *δ* 7.56–7.53 (m, 1H), 7.50 (dd, *J* = 7.2, 1.8 Hz, 2H), 7.35–7.22 (m, 3H), 6.52 (d, *J* = 3.3 Hz, 1H), 6.40 (dd, *J* = 3.3, 1.9 Hz, 1H), 5.45 (s, 1H), 3.59 (q, *J* = 5.3 Hz, 4H), 2.78–2.62 (m, 4H) ppm. ^13^C NMR (101 MHz, Acetone-*d_6_*): *δ* 152.9, 143.5, 136.9, 133.2, 129.8, 127.8, 111.0, 110.5, 76.2, 67.2, 50.0 ppm. m.p. = 50–52 °C. HRMS (*m*/*z*) calcd for C_15_H_17_NO_2_S ([M+H]^+^) 276.10528, found 276.10445.

*4-(phenyl(phenylthio)methyl)morpholine* (**2**). Prepared according to general procedure using benzaldehyde (0.1054 g, 0.993 mmol, 1 equiv), morpholine (0.0887 g, 1.02 mmol, 1.03 equiv) and thiophenol (0.1084 g, 0.984 mmol, 0.99 equiv). The compound was obtained as a pale-yellow solid (0.2072 g, 76% yield). ^1^H NMR (300 MHz, Acetone-*d_6_*): *δ* 7.65–7.61 (m, 2H), 7.47–7.44 (m, 2H), 7.40–7.21 (m, 6H), 5.53 (s, 1H), 3.64–3.53 (m, 4H), 2.75–2.60 (m, 4H) ppm [39]. ^13^C NMR (75 MHz, Acetone-*d_6_*): *δ* 139.9, 137.7, 132.8, 129.6, 128.9, 128.3, 128.0, 127.5, 81.7, 67.3, 50.3. m.p. = 41–43 °C (lit. 35–36 °C) [40]. HRMS (*m*/*z*) calcd for C_17_H_19_NOS ([M+H]^+^) 286.12601, found 286.12766.

*4-((phenylthio)(p-tolyl)methyl)morpholine* (**3**). Prepared according to general procedure using 4-methylbenzaldehyde (0.1311 g, 1.09 mmol, 1 equiv), morpholine (0.11312 g, 1.3 mmol, 1.19 equiv) and thiophenol (0.1038 g, 0.942 mmol, 0.86 equiv). The compound was obtained as a pale-yellow solid (0.2371 g, 84% yield). ^1^H NMR (300 MHz, Acetone-*d_6_*): *δ* 7.52–7.49 (m, 2H), 7.46–7.43 (m, 2H), 7.29–7.22 (m, 2H), 7.22–7.16 (m, 3H), 5.50 (s, 1H), 3.61–3.55 (m, 4H), 2.70–2.62 (m, 4H), 2.32 (s, 3H) ppm. ^13^C NMR (101 MHz, Acetone-*d_6_*): *δ* 139.2, 137.5, 135.7, 132.8, 130.2, 129.7, 129.7, 128.5, 128.2, 89.5, 82.9, 68.1, 67.6, 67.5, 67.3, 65.7, 51.1, 50.4, 50.2, 48.0, 46.9, 21.0 ppm. m.p. = 44–47 °C. HRMS (*m*/*z*) calcd for C_18_H_21_NOS ([M+H]^+^) 300.14166, found 300.14304.

*4-((phenylthio)(4-(trifluoromethyl)phenyl)methyl)morpholine* (**4**). Prepared according to general procedure using 4-(trifluoromethyl)benzaldehyde (0.1634 g, 0.938 mmol, 1 equiv), morpholine (0.0835 g, 0.958 mmol, 1.02 equiv) and thiophenol (0.1308 g, 1.19 mmol, 1.27 equiv). The compound was obtained as a pale-orange solid (0.2489 g, 75% yield). ^1^H NMR (300 MHz, Acetone-*d_6_*): δ 7.91–7.87 (m, 2H), 7.74–7.70 (m, 2H), 7.50–7.46 (m, 2H), 7.36–7.21 (m, 3H), 5.62 (s, 1H), 3.69–3.55 (m, 4H), 2.77–2.63 (m, 4H) ppm. ^13^C NMR (101 MHz, Acetone-*d_6_*): *δ* 144.7, 136.9, 133.0, 130.4, 130.0, 129.2 (q), 127.9, 127.2 (q), 125.86 (q), 82.5, 67.2, 50.4 ppm. m.p. = 63–64 °C. HRMS (*m*/*z*) calcd for C_18_H_18_F_3_NOS ([M+H]^+^) 354.11340, found 354.11067.

*4-((4-bromophenyl)(phenylthio)methyl)morpholine* (**5**). Prepared according to general procedure using 4-bromobenzaldehyde (0.1728 g, 0.934 mmol, 1 equiv), morpholine (0.0821 g, 0.942 mmol, 1.01 equiv) and thiophenol (0.104 g, 0.942 mmol, 1.01 equiv). The compound was obtained as a pale-yellow solid (0.2843 g, 84% yield). ^1^H NMR (400 MHz, Acetone-*d_6_*): *δ* 7.61 (d, *J* = 8.5 Hz, 2H), 7.55 (d, *J* = 8.5 Hz, 3H), 7.47–7.43 (m, 2H), 7.27 (dd, *J* = 7.1, 3.4 Hz, 3H), 5.52 (s, 1H), 3.65 – 3.54 (m, 6H), 2.74–2.60 (m, 4H) ppm. ^13^C NMR (101 MHz, Acetone-*d_6_*): *δ* 139.5, 137.2, 132.9, 132.0, 131.8, 130.2, 129.9, 128.2, 127.7, 122.1, 82.4, 67.3, 50.3 ppm. m.p. = 58–60 °C. HRMS (*m*/*z*) calcd for C_17_H_18_BrNOS ([M+H]^+^) 364.03652, found 364.03788.

*1-(phenyl(phenylthio)methyl)piperidine* (**6**). Prepared according to general procedure using benzaldehyde (0.100 g, 0.972 mmol, 1 equiv), piperidine (0.0802 g, 0.942 mmol, 1 equiv) and thiophenol (0.1038 g, 0.942 mmol, 1 equiv). The compound was obtained as a pale-yellow oil (0.2307 g, 86% yield). ^1^H NMR (300 MHz, Acetone-*d_6_*): *δ* 7.69–7.63 (m, 2H), 7.48–7.41 (m, 2H), 7.38–7.21 (m, 7H), 5.57 (s, 1H), 2.65 (d, *J* = 17.4 Hz, 4H), 1.57–1.39 (m, 7H) ppm. ^13^C NMR (101 MHz, Acetone-*d_6_*): *δ* 140.7, 137.3, 133.0, 132.4, 130.1, 129.7, 129.5, 128.7, 128.6, 128.1, 84.5, 50.9, 26.6, 25.0 ppm. HRMS (*m*/*z*) calcd for C_18_H_21_NS ([M+H]^+^) 284.14675, found 284.14743.

*1-methyl-4-(phenyl(phenylthio)methyl)piperazine* (**7**). Prepared according to general procedure using benzaldehyde (0.100 g, 0.942 mmol, 1 equiv), *N*-methyl piperazine (0.0944 g, 0.942 mmol, 1 equiv) and thiophenol (0.1038 g, 0.942 mmol, 1 equiv). The compound was obtained as a hygroscopic orange solid (0.1852 g, 66% yield). ^1^H NMR (400 MHz, Acetone-*d_6_*): *δ* 7.67–7.61 (m, 2H), 7.56–7.52 (m, 1H), 7.47–7.42 (m, 2H), 7.38–7.33 (m, 3H), 7.28 (dt, *J* = 10.6, 7.6 Hz, 4H), 5.59 (s, 1H), 2.76 – 2.63 (m, 4H), 2.34 (s, 5H), 2.16 (s, 3H) ppm. ^13^C NMR (101 MHz, Acetone-*d_6_*): *δ* 140.3, 137.4, 132.6, 130.2, 129.7, 129.6, 128.8, 128.5, 128.3, 128.2, 127.3, 83.1, 55.8, 46.3 ppm. m.p. = low melting point. HRMS (*m*/*z*) calcd for C_18_H_22_N_2_S ([M+H]^+^) 299.15765, found 299.16097.

*N,N-dibenzyl-1-phenyl-1-(phenylthio)methanamine* (**8**). Prepared according to general procedure using benzaldehyde (0.100 g, 0.942 mmol, 1 equiv), dibenzylamine (0.1859 g, 0.942 mmol, 1 equiv) and thiophenol (0.1038 g, 0.942 mmol, 1 equiv). The compound was obtained as a pale-yellow oil (0.3547 g, 95% yield). ^1^H NMR (300 MHz, Acetone-*d*_6_): *δ* 7.82–7.76 (m, 2H), 7.42–7.37 (m, 2H), 7.33–7.19 (m, 17H), 5.48 (s, 1H), 3.79 (s, 4H) ppm. ^13^C NMR (101 MHz, Acetone-*d_6_*): δ 140.9, 139.6, 137.6, 132.7, 129.9, 129.8, 129.7, 129.3, 129.2, 129.01, 129.0, 128.7, 128.1, 127.4, 79.0, 54.4 ppm. HRMS (*m*/*z*) calcd for C_27_H_25_NS ([M+H]^+^) 396.17805, found 396.17722.

*4-(phenyl(p-tolylthio)methyl)morpholine* (**9**). Prepared according to general procedure using benzaldehyde (0.0997 g, 0.939 mmol, 1 equiv), morpholine (0.0833 g, 0.956 mmol, 1.02 equiv) and 4-methylbenzenethiol (0.1198 g, 0.965 mmol, 1.03 equiv). The compound was obtained as a pale-yellow solid (0.2097 g, 75% yield). ^1^H NMR (300 MHz, Acetone-*d_6_*): *δ* 7.63–7.60 (m, 2H), 7.42–7.29 (m, 5H), 7.09 (d, *J* = 7.8 Hz, 2H), 5.43 (s, 1H), 3.61–3.56 (m, 4H), 2.72–2.62 (m, 4H), 2.27 (s, 3H) ppm. ^13^C NMR (101 MHz, Acetone-*d_6_*): *δ* 140.0, 137.5, 133.9, 133.3, 130.5, 129.6, 128.9, 128.5, 83.5, 67.3, 50.3, 21.0 ppm. m.p. = 45–47 °C. HRMS (*m*/*z*) calcd for C_18_H_21_NOS ([M+H]^+^) 300.14166, found 300.14249.

*4-(((3,5-dimethylphenyl)thio)(phenyl)methyl)morpholine* (**10**). Prepared according to general procedure using benzaldehyde (0.1008 g, 0.950 mmol, 1 equiv), morpholine (0.083 g, 0.953 mmol, 1 equiv) and 3,5-dimethylbenzenethiol (0.1295 g, 0.937 mmol, 0.99 equiv). The compound was obtained as a pale-yellow solid (0.190 g, 67% yield). ^1^H NMR (300 MHz, Acetone-*d_6_*): *δ* 7.65–7.62 (m, 2H), 7.39–7.27 (m, 4H), 7.10–7.09 (m, 2H), 5.52 (s, 1H), 3.69–3.53 (m, 4H), 2.74–2.59 (m, 4H), 2.22 (m, 6H) ppm. ^13^C NMR (101 MHz, Acetone-*d_6_*): *δ* 140.1, 139.1, 137.3, 130.2, 129.6, 129.2, 128.9, 125.8, 82.9, 67.3, 50.3, 21.2 ppm. m.p. = 37–39 °C. HRMS (*m*/*z*) calcd for C_19_H_23_NOS ([M+H]^+^) 314.15731, found 314.16031.

*4-(((4-methoxyphenyl)thio)(phenyl)methyl)morpholine* (**11**). Prepared according to general procedure using benzaldehyde (0.1014 g, 0.955 mmol, 1 equiv), morpholine (0.0939 g, 1.08 mmol, 1.13 equiv) and 4-methoxybenzenethiol (0.1326 g, 0.946 mmol, 0.99 equiv). The compound was obtained as a pale-yellow solid (0.2089 g, 86% yield). ^1^H NMR (300 MHz, Acetone-*d_6_*): *δ* 7.59–7.57 (m, 2H), 7.41–7.25 (m, 5H), 6.87–6.84 (m, 2H), 5.29 (s, 1H), 3.77 (s, 3H), 3.65–3.52 (m, 4H), 2.75–2.68 (m, 2H), 2.65–2.59 (m, 2H) ppm. ^13^C NMR (75 MHz, Acetone- *d_6_*): *δ* 160.3, 140.1, 135.9, 129.6, 128.8, 128.5, 127.4, 115.4, 84.1, 67.4, 55.6, 50.5 ppm. m.p. = 69–71 °C. HRMS (*m*/*z*) calcd for C_18_H_21_NO_2_S ([M+H]^+^) 316.13658, found 316.13639.

*4-(((3,4-dimethoxyphenyl)thio)(phenyl)methyl)morpholine* (**12**). Prepared according to general procedure using benzaldehyde (0.1003 g, 0.945 mmol, 1 equiv), morpholine (0.0853 g, 0.979 mmol, 1.04 equiv) and 3,5-dimethoxybenzenethiol (0.159 g, 0.934 mmol, 0.99 equiv). The compound was obtained as a pale-yellow solid (0.187 g, 58% yield). ^1^H NMR (300 MHz, Acetone-*d*_6_): *δ* 7.59–7.56 (m, 2H), 7.38–7.28 (m, 3H), 7.02–6.99 (m, 2H), 6.86–6.83 (m, 1H), 5.35 (s, 1H), 3.77 (s, 1H), 3.74 (s, 3H), 3.62–3.57 (m, 4H), 2.73–2.64 (m, 4H). ^13^C NMR (101 MHz, Acetone-*d_6_*): *δ* 206.2, 151.0, 150.6, 140.1, 130.3, 129.6, 128.8, 128.4, 126.9, 124.4, 115.0, 112.9, 83.7, 67.4, 56.1, 56.1, 50.5 ppm. m.p. = 74–78 °C. HRMS (*m*/*z*) calcd for C_19_H_23_NO_3_S ([M+H]^+^) 346.14714, found 346.14753.

*4-(((4-fluorophenyl)thio)(phenyl)methyl)morpholine* (**13**). Prepared according to general procedure using benzaldehyde (0.1066 g, 1.0 mmol, 1 equiv), morpholine (0.0916 g, 1.05 mmol, 1.05 equiv) and 4-fluorobenzenethiol (0.1283 g, 1.0 mmol, 1 equiv). The compound was obtained as a pale-yellow solid (0.2065 g, 70% yield). ^1^H NMR (300 MHz, Acetone-*d_6_*): *δ* 7.61–7.56 (m, 2H), 7.53–7.46 (m, 2H), 7.39–7.30 (m, 3H), 7.09–7.02 (t, *J* = 8.8 Hz, 2H), 5.44 (s, 1H), 3.61–3.56 (m, 5H), 2.70–2.64 (m, 4H) ppm. ^13^C NMR (101 MHz, Acetone-*d_6_*): *δ* 164.7, 162.3, 139.5, 132.9, 132.1, 132.0, 129.6, 128.9, 128.5, 117.3, 117.1, 83.6, 67.5, 50.4 ppm. m.p. = 47–49 °C. HRMS (*m*/*z*) calcd for C_17_H_18_FNOS ([M+H]^+^) 304.11659, found 304.11812.

*4-(((4-chlorophenyl)thio)(phenyl)methyl)morpholine* (**14**). Prepared according to general procedure using benzaldehyde (0.100 g, 0.942 mmol, 1 equiv), morpholine (0.0869 g, 0.997 mmol, 1.06 equiv) and 4-chlorobenzenethiol (0.1369 g, 0.947 mmol, 1 equiv). The compound was obtained as a pale-yellow solid (0.2243 g, 74% yield). ^1^H NMR (300 MHz, Acetone-*d_6_*): *δ* 7.62–7.59 (m, 2H), 7.47–7.44 (m, 2H), 7.40–7.28 (m, 5H), 5.55 (s, 1H), 3.59 (q, *J* = 5.2 Hz, 4H), 2.67 (q, *J* = 5.1 Hz, 4H) ppm. ^13^C NMR (75 MHz, Acetone- *d_6_*): *δ* 139.4, 136.6, 134.3, 133.0, 129.8, 129.6, 129.0, 128.8, 83.1, 67.3, 50.3 ppm. m.p. = 87–88 °C. HRMS (*m*/*z*) calcd for C_17_H_18_ClNOS ([M+H]^+^) 320.08704, found 320.09261.

*4-(((3,5-dichlorophenyl)thio)(phenyl)methyl)morpholine* (**15**). Prepared according to general procedure using benzaldehyde (0.1039 g, 0.979 mmol, 1 equiv), morpholine (0.0891 g, 1.02 mmol, 1.04 equiv) and 3,5-dichlorobenzenethiol (0.1751 g, 0.978 mmol, 1 equiv). The compound was obtained as a pale-yellow solid (0.2548 g, 91% yield). ^1^H NMR (300 MHz, Acetone-*d_6_*): *δ* 7.62–7.60 (m, 2H), 7.42–7.29 (m, 6H), 5.80 (s, 1H), 3.61–3.58 (m, 4H), 2.72–2.68 (m, 4H) ppm. ^13^C NMR (75 MHz, Acetone-*d_6_*): *δ* 140.2, 138.6, 135.5, 130.0, 129.6, 129.3, 129.1, 129.0, 126.9, 82.2, 67.3, 50.1 ppm. M.p. = 97–100 °C. HRMS (*m*/*z*) calcd for C_17_H_17_Cl_2_NOS ([M+H]^+^) 354.04807, found 354.05168.

*4-((benzylthio)(phenyl)methyl)morpholine* (**16**). Prepared according to general procedure using benzaldehyde (0.1025 g, 0.966 mmol, 1 equiv), morpholine (0.0865 g, 0.993 mmol, 1.03 equiv) and phenylmethanethiol (0.1208 g, 0.973 mmol, 1.01 equiv). The compound was obtained as a pale-orange oil (0.2278 g, 79% yield). ^1^H NMR (300 MHz, Acetone-*d_6_*): *δ* 7.41 (m, 2H), 7.38–7.26 (m, 8H), 4.85 (s, 1H), 3.90 (d, *J* = 13.1 Hz, 1H), 3.71 (d, *J* = 13.1 Hz, 1H), 3.61 (t, *J* = 4.7 Hz, 4H), 2.64–2.57 (m, 2H), 2.53–2.46 (m, 2H) ppm. ^13^C NMR (101 MHz, Acetone-*d_6_*): *δ* 139.8, 138.3, 129.9, 129.5, 129.2, 128.4, 128.6, 127.6, 75.8, 67.5, 50.0, 36.0 ppm. HRMS (*m*/*z*) calcd for C_18_H_21_NOS ([M+H]^+^) 300.14166, found 300.14211.

*4-((phenethylthio)(phenyl)methyl)morpholine* (**17**). Prepared according to general procedure using benzaldehyde (0.1017 g, 0.958 mmol, 1 equiv), morpholine (0.0923 g, 1.06 mmol, 1.10 equiv) and 2-phenylethane-1-thiol (0.1315 g, 0.951 mmol, 1 equiv). The compound was obtained as a pale-yellow oil (0.2697 g, 90% yield). ^1^H NMR (300 MHz, Acetone-*d_6_*): *δ* 7.48–7.45 (m, 2H), 7.38–7.22 (m, 8H), 4.97 (s, 1H), 3.60 (t, *J* = 4.7 Hz, 4H), 2.90–2.88 (m, 3H), 2.77–2.73 (m, 1H), 2.64–2.50 (m, 4H) ppm. ^13^C NMR (101 MHz, Acetone-*d_6_*): *δ* 141.8, 139.0, 129.5, 129.4, 129.2, 129.1, 128.8, 128.5, 127.0, 76.8, 67.5, 67.3, 50.2, 37.3, 33.6 ppm. HRMS (*m*/*z*) calcd for C_19_H_23_NOS ([M+H]^+^) 314.15731, found 314.15854.

*4-(phenyl(propylthio)methyl)morpholine* (**18**). Prepared according to general procedure using benzaldehyde (0.100 g, 0942 mmol, 1 equiv), morpholine (0.082 g, 0.942 mmol, 1 equiv) and propanethiol (0.0718 g, 0.942 mmol, 1 equiv). The compound was obtained as a pale-yellow oil (0.1687 g, 71% yield). ^1^H NMR (400 MHz, Acetone-*d_6_*): *δ* 7.52–7.48 (m, 2H), 7.37–7.32 (m, 2H), 7.31–7.27 (m, 1H), 4.95 (s, 1H), 3.65–3.54 (m, 6H), 2.58 (dt, *J* = 10.9, 5.7 Hz, 5H), 2.48 (dt, *J* = 12.6, 7.3 Hz, 2H), 1.59 (m, 2H), 0.95 (t, *J* = 7.3 Hz, 3H) ppm. ^13^C NMR (101 MHz, Acetone-*d_6_*): *δ* 139.4, 129.4, 128.7, 128.3, 77.1, 67.4, 50.2, 34.3, 23.9, 13.8 ppm. HRMS (*m/z*) calcd for C_14_H_21_NOS ([M+H]^+^) 252.14166, found 252.13803.

*4-((dodecylthio)(phenyl)methyl)morpholine* (**19**). Prepared according to general procedure using benzaldehyde (0.1 g, 0.942 mmol, 1 equiv), morpholine (0.0821 g, 0.942 mmol, 1 equiv) and dodecanethiol (0.191 g, 0.942 mmol, 1 equiv). The compound was obtained as a white solid (0.3468 g, 97% yield). ^1^H NMR (300 MHz, Acetone-*d_6_*): *δ* 7.52–7.46 (m, 2H), 7.39–7.27 (m, 3H), 4.96 (s, 1H), 3.60 (dd, J = 5.3, 4.0 Hz, 5H), 2.62–2.55 (m, 5H), 1.58 (dq, J = 8.3, 7.0 Hz, 2H), 1.28 (s, 21H), 0.90–0.85 (m, 3H) ppm. ^13^C NMR (101 MHz, Acetone-*d_6_*): 139.5, 129.5, 128.4, 77.1, 67.5, 50.3, 32.6, 32.2, 30.7, 30.4, 30.3, 30.2, 30.1, 29.9, 29.6, 23.3, 14.4 ppm. m.p. = 52–54 °C. HRMS (*m*/*z*) calcd for C_23_H_39_NOS ([M+H]^+^) 378.28521, found 378.28370.

### 3.4. General Procedure for the Thiol Exchange

Aminal **20** (0.100 g, 0.381 mmol) was dissolved in a freshly prepared solution of Cu(OTf)_2_ (0.00138 g, 0.01 equiv) in acetonitrile (1.9 mL). Thiophenol (0.042 g, 0.381 mmol) was added and the mixture was allowed to react for 10 min. The crude was evaporated under reduced pressure and the obtained solid was washed with cold distilled water (5 mL), yielding thioaminal **2** as a pale-yellow solid (0.102 g, 94% yield).

### 3.5. General Procedure for UV Stabilities Experiments of Thioaminals **9**, **11** and **14**

Thioaminals were dissolved in MeOH (10 mM) and diluted in a solution of MeCN or ammonium acetate 20 mM (pH 7) buffer at 0.01 mM.

Then, 10 mM solutions of aminals **9, 11** and **14** in methanol were freshly prepared and were diluted in 2 mL of acetonitrile or ammonium acetate 20 mM (pH 7) buffer to a final concentration of 100 µM. The quartz cuvette was swiftly mixed by inversion and analyzed by UV-Visible spectroscopy. Full-scan analyses were performed every minute until a constant absorbance value was reached. To compare the results obtained, we plotted the absorbance values obtained for each thioaminal at a particular wavelength over time (250 nm).

## 4. Conclusions

The preparation of *N*,*S*-thioaminals is an ongoing challenge, due to both the reactivity and the stability of the products. Previous conditions required high temperatures and/or hazardous reagents. In accordance with the principles of green chemistry, in this work we expanded our previously reported conditions to thioaminals, affording the products under mild conditions, in aqueous media promoted by catalytic amounts of base metal copper(II) triflate. We hope that this report will contribute to the valorization of the thioaminal scaffold, both for drug discovery but also as a synthon for further derivatizations.

## Figures and Tables

**Table 1:** Effect of Cu(OTf)_2_ on the formation of thioaminals in aqueous media. ^a^ isolated yield. ^b^ Yield based on crude NMR.

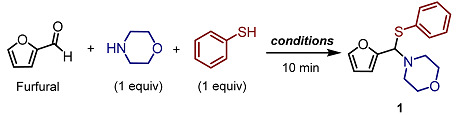				
Entry	Catalyst	Solvent	Conversion (%)	Yield of 1 (%)
1	no catalyst	H_2_O	0	0
2	Cu(OTf)_2_ (10 mol%)	H_2_O	100	0
**3**	**Cu(OTf)_2_ (1 mol%)**	**H_2_O**	**100**	**100 (84)^a^**
4	Cu(OTf)_2_ (1 mol%)	neat	93	66^a,b^
5	Cu(OTf)_2_ (1 mol%)	EtOH	64	29^a,b^
6	Cu(OTf)_2_ (1 mol%)	MeCN	93	56^a,b^
7	CuSO_4_ (1 mol%)	H_2_O	100	35
8	FeCl_3_ (1 mol%)	H_2_O	100	0
9	AlCl_3_ (1 mol%)	H_2_O	100	12
10	ZnCl_2_ (1 mol%)	H_2_O	100	70
11	TfOH (2 mol%)	H_2_O	87	40

## Data Availability

Electronic supplementary information is given, including all NMR spectra and additional experimental details regarding UV stabilities studies.

## Supplementary Materials

The following supporting information can be downloaded online. Figures S1–S20: ^1^H NMR, ^13^C NMR of compounds **1**-**19**. Figure S21: Variation of UV spectrum of thioaminal 9 after 20 s at pH 7; Figure S22: Variation of UV spectrum of thioaminal 11 after 20 s at pH 7; Figure S23: Variation of UV spectrum of thioaminal 14 after 20 s at pH 7. Reference [41] is Cited in Supplementary Materials.

## Sample Availability

Samples of the compounds are not available from the authors.
