# Supplementary material for: Preparation of Thioaminals in Water
Source: Molecules. 2022 Mar 3;27(5):1673. doi: 10.3390/molecules27051673 (PMC8911761; doi:10.3390/molecules27051673)
Supplement: Supplementary file 1 [file molecules-27-01673-s001.zip › molecules-1565115-SI_Final.pdf]

molecules

MDPI

Supporting Information

Preparation of thioaminals in water

Lídia A. S. Cavaca, Rafael F. A. Gomes* and Carlos A. M. Afonso*

Research Institute for Medicines (iMed.ULisboa), Faculty of Pharmacy, University of Lisbon, Av. Prof. Gama Pinto, 1649-003 Lisboa, Portugal; l.cavaca@campus.fct.unl.pt

* Correspondence: rafael.gomes@campus.ul.pt (R.F.A.G.); carlosafonso@ff.ulisboa.pt (C.A.M.A.)

Preparation of starting materials

Synthesis of 1-(piperazin-1-yl)ethan-1-one

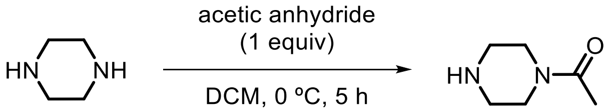

Procedure according to literature[1]: Piperazine (1.7 g, 19.74 mmol, 2 equiv) was dissolved in DCM (50 mL). The solution was cooled to 0 °C, followed by dropwise addition of acetic anhydride (0.940 mL, 9.94 mmol, 1 equiv). The reaction was allowed to stir at 0 °C for 5 hours. The reaction mixture was quenched with distilled H_2O (15 mL). The organic layer was dried with $MgSO_4$ (anhyd), filtered and evaporated under reduced pressure to yield a white solid containing a mixture of monoacetylated and diacetylated piperazine in about 10:1 ratio. Monoacetylated piperazine was obtained after purification by column chromatography in silica DCM/MeOH (9:1) then DCM/MeOH (1:1). After evaporation of the desired fractions, the compound was redissolved in DCM, filtered and the solvent evaporated to yield the product as a yellow solid (0.0343g, 1.4% yield).

^{1}H NMR (300 MHz, $CDCl_3$): δ 3.51 (t, J = 5.2 Hz, 2H), 3.37 (t, J = 5.2 Hz, 2H), 2.78 (dt, J = 10.7, 4.7 Hz, 4H), 2.02 (d, J = 1.7 Hz, 3H) ppm.

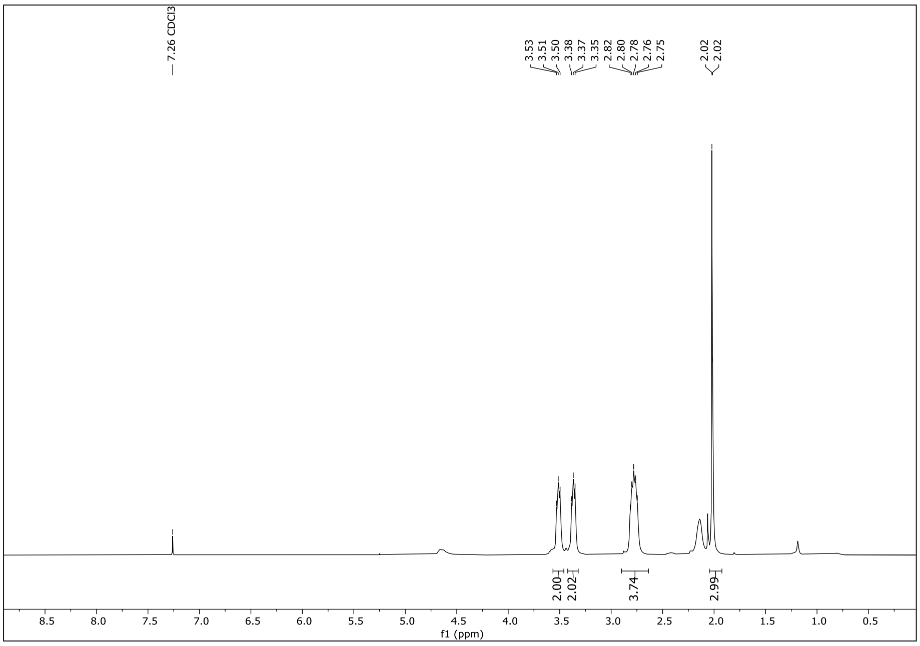

Figure S1. ^{1}H-NMR Spectra of 1-(piperazin-1-yl)ethan-1-one.

NMR spectra of thioaminals

*4-(furan-2-yl(phenylthio)methyl)morpholine (**1**)*

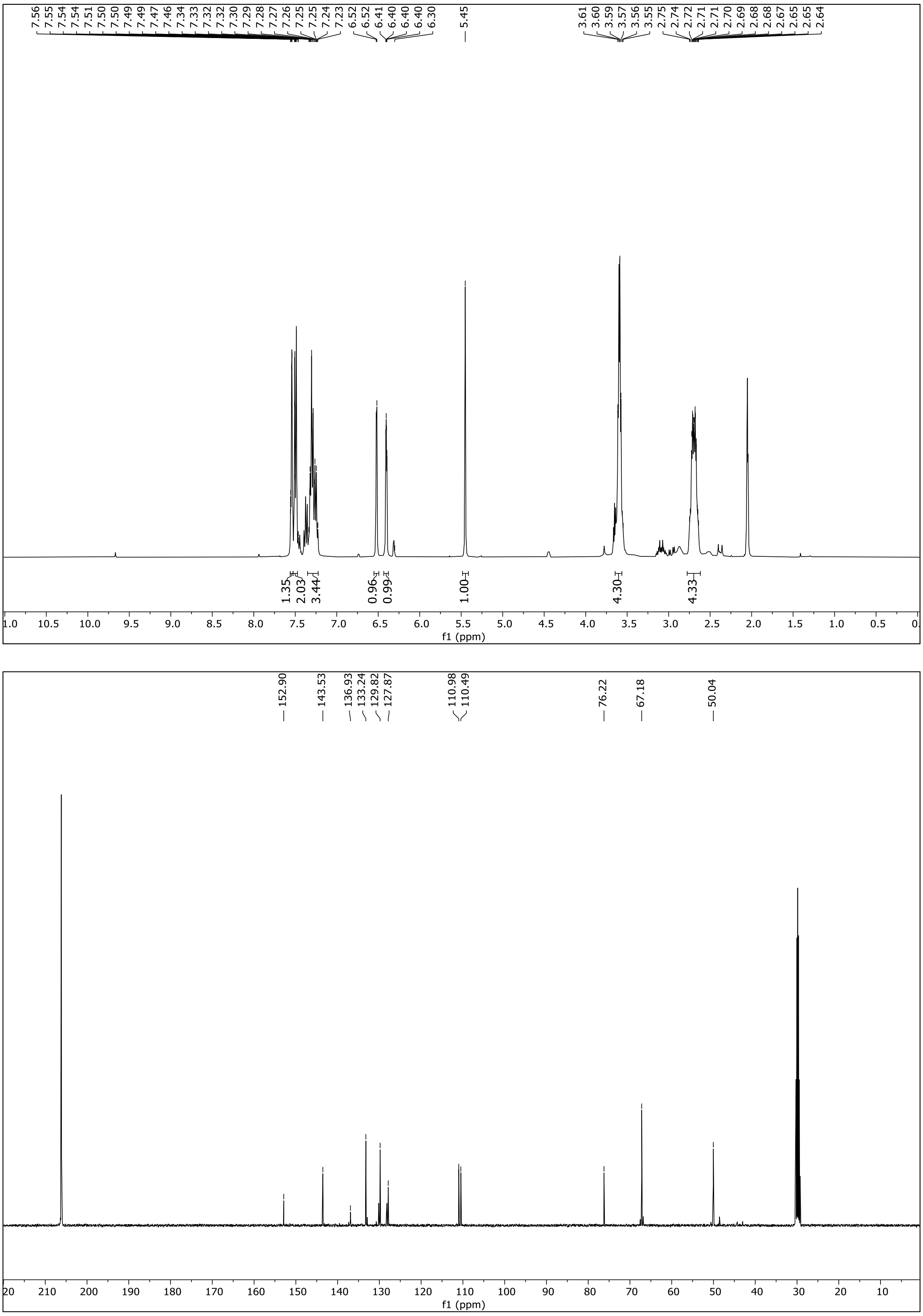

Figure S2. ^{1}H-NMR and ^{13}C-NMR spectra of **1**.

4-(phenyl(phenylthio)methyl)morpholine (**2**)

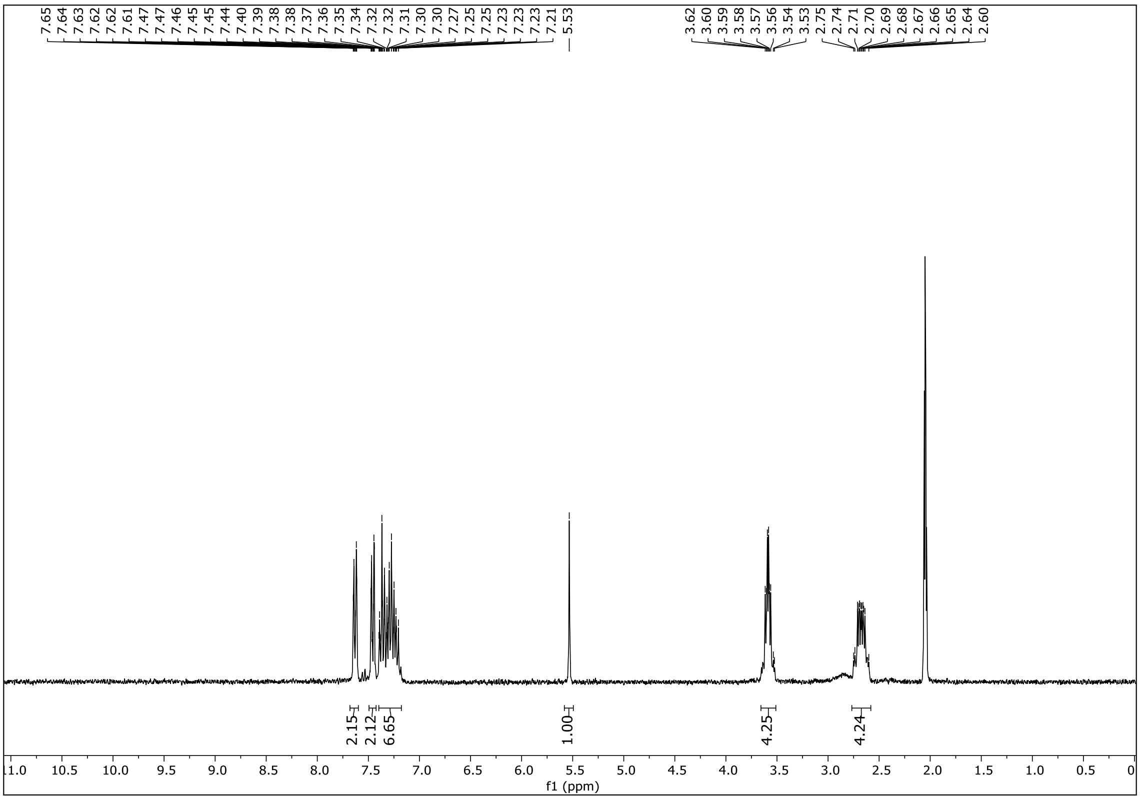

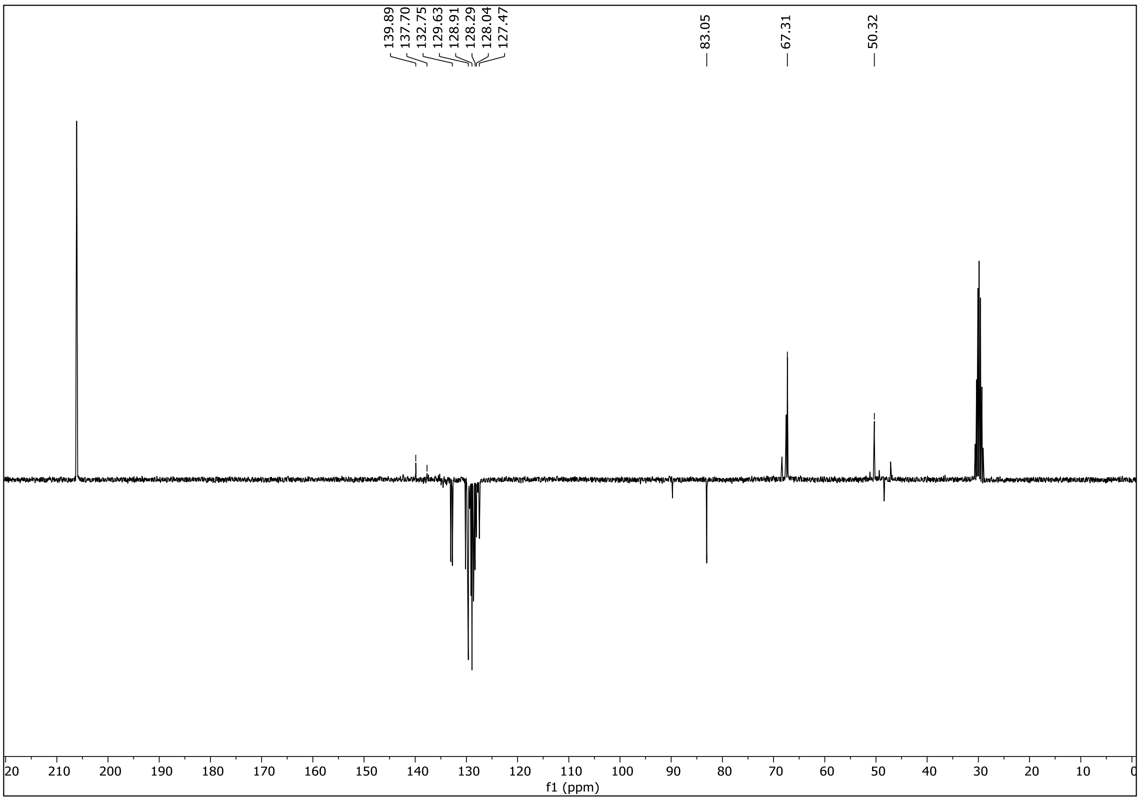

Figure S3. ^{1}H-NMR and ^{13}C-NMR spectra of **2.**

*4-((phenylthio)(p-tolyl)methyl)morpholine (**3**)*

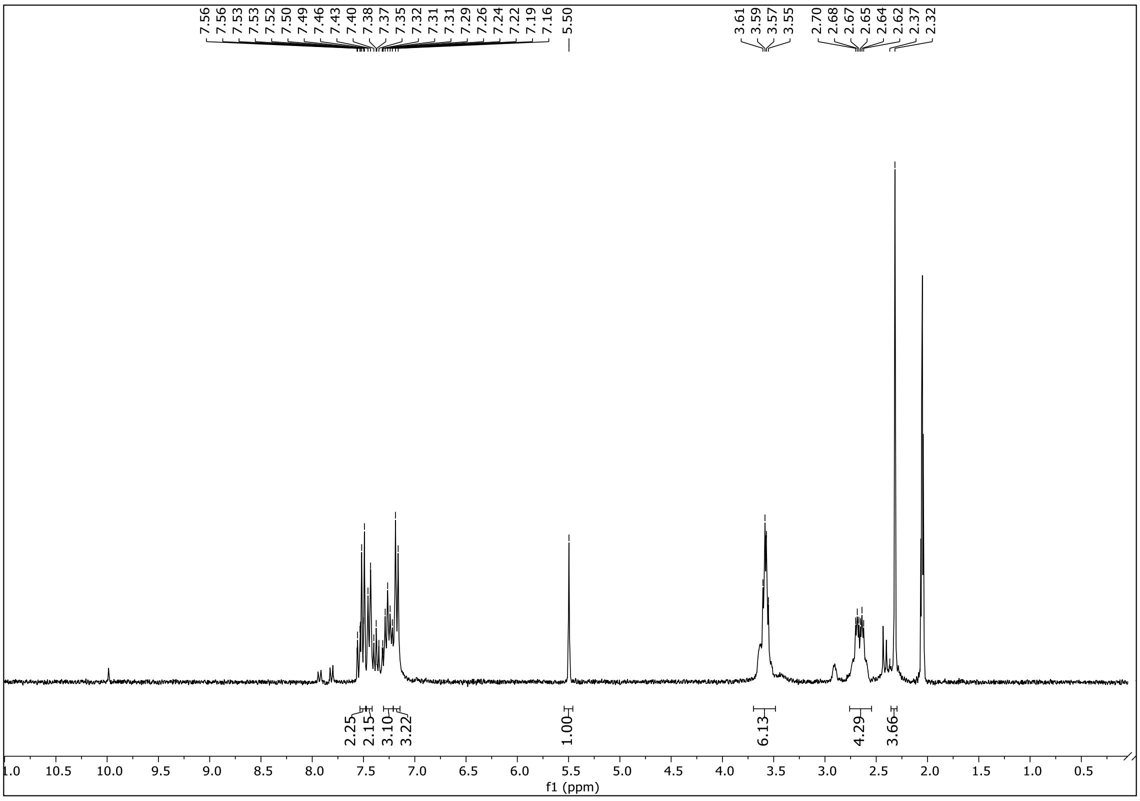

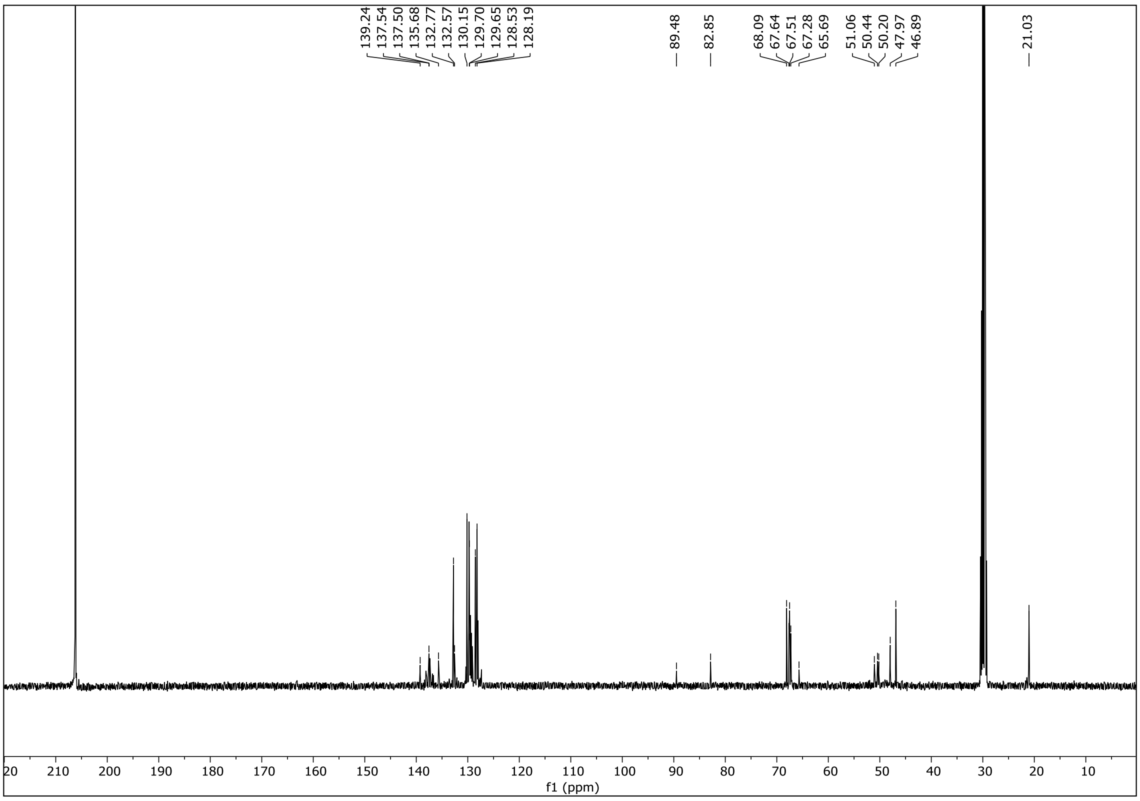

Figure S4. ^{1}H-NMR and ^{13}C-NMR spectra of **3**.

*4-((phenylthio)(4-(trifluoromethyl)phenyl)methyl)morpholine (**4**)*

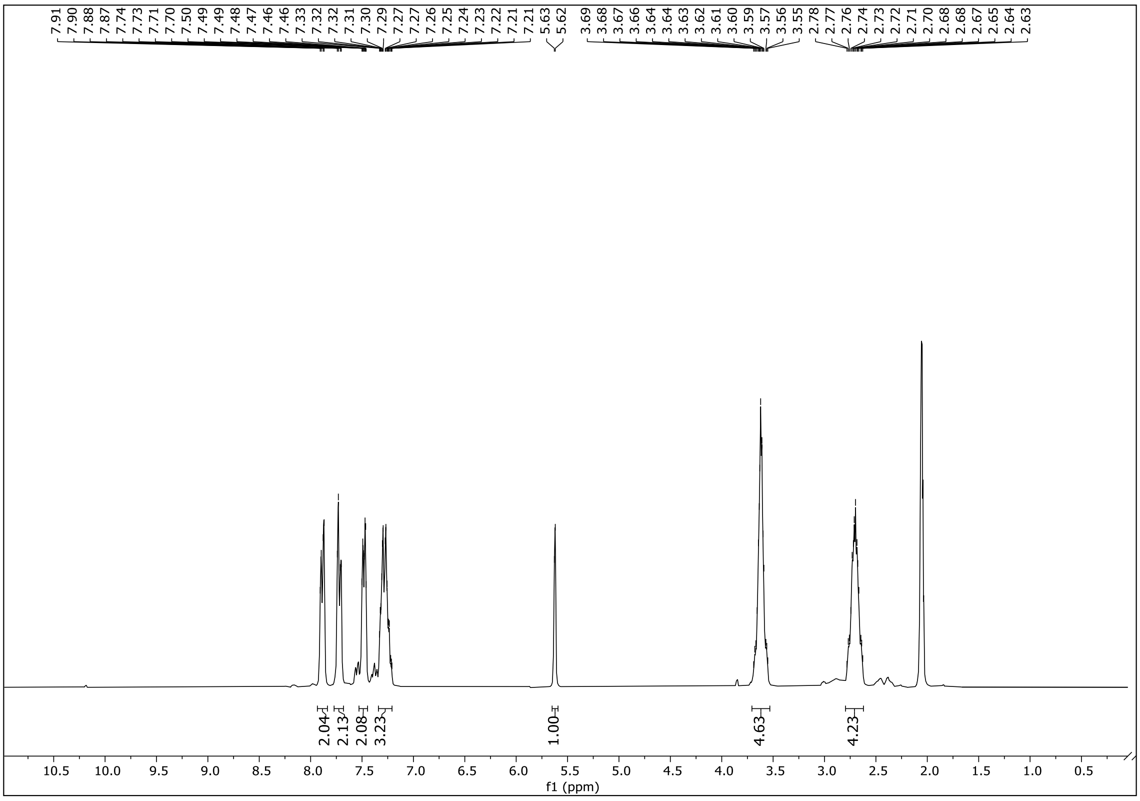

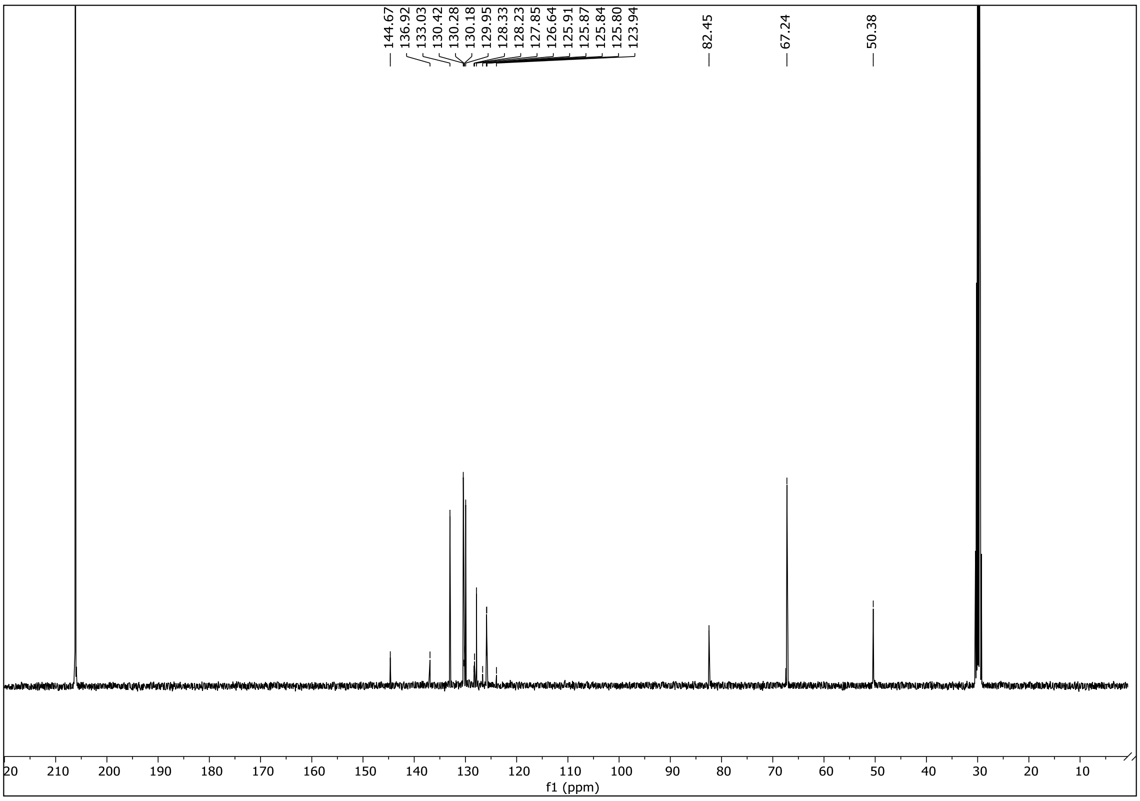

Figure S5. ^{1}H-NMR and ^{13}C-NMR spectra of **4**.

*4-((4-bromophenyl)(phenylthio)methyl)morpholine (**5**)*

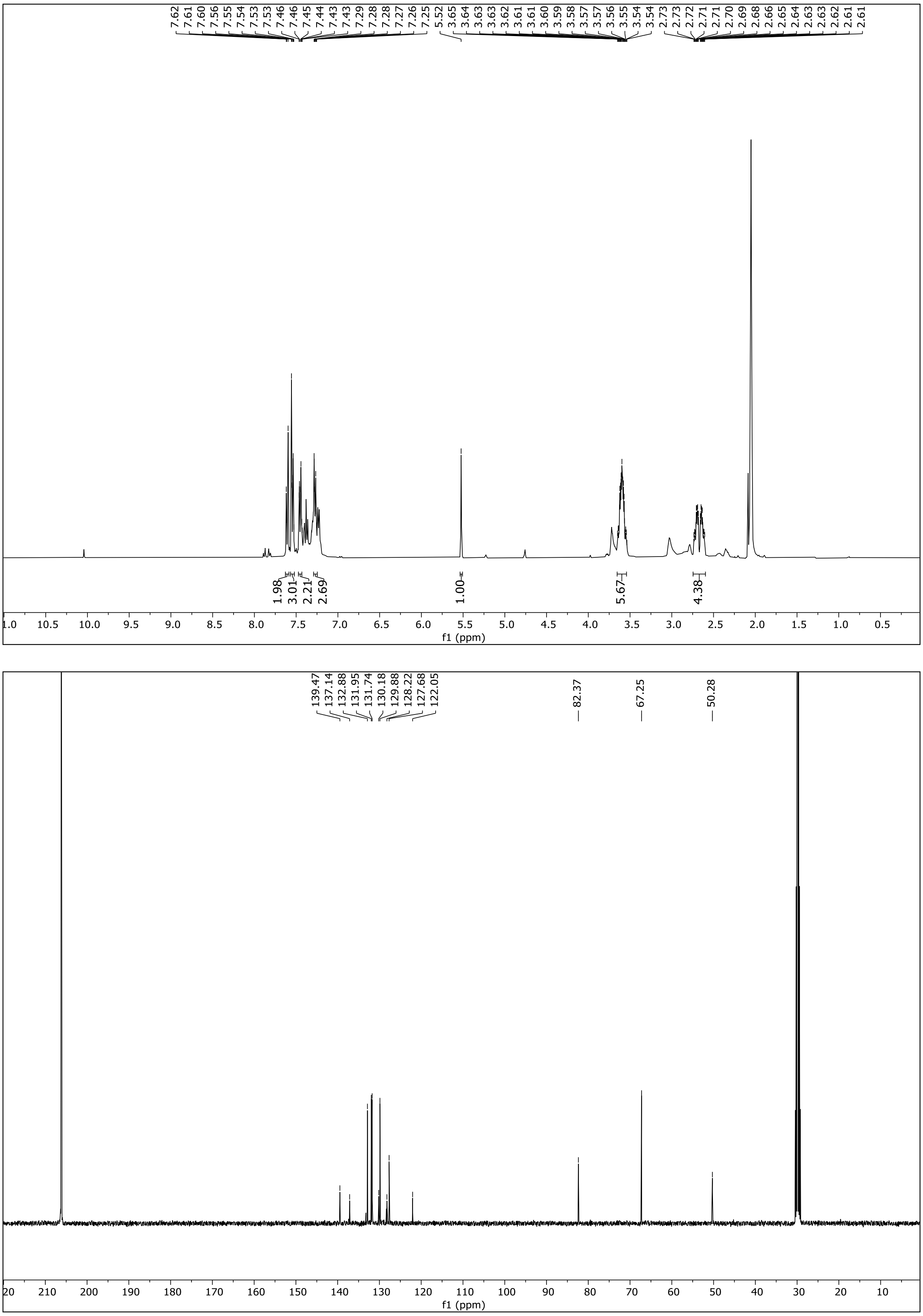

Figure S6. ^{1}H-NMR and ^{13}C-NMR spectra of **5**.

*1-(phenyl(phenylthio)methyl)piperidine (**6**)*

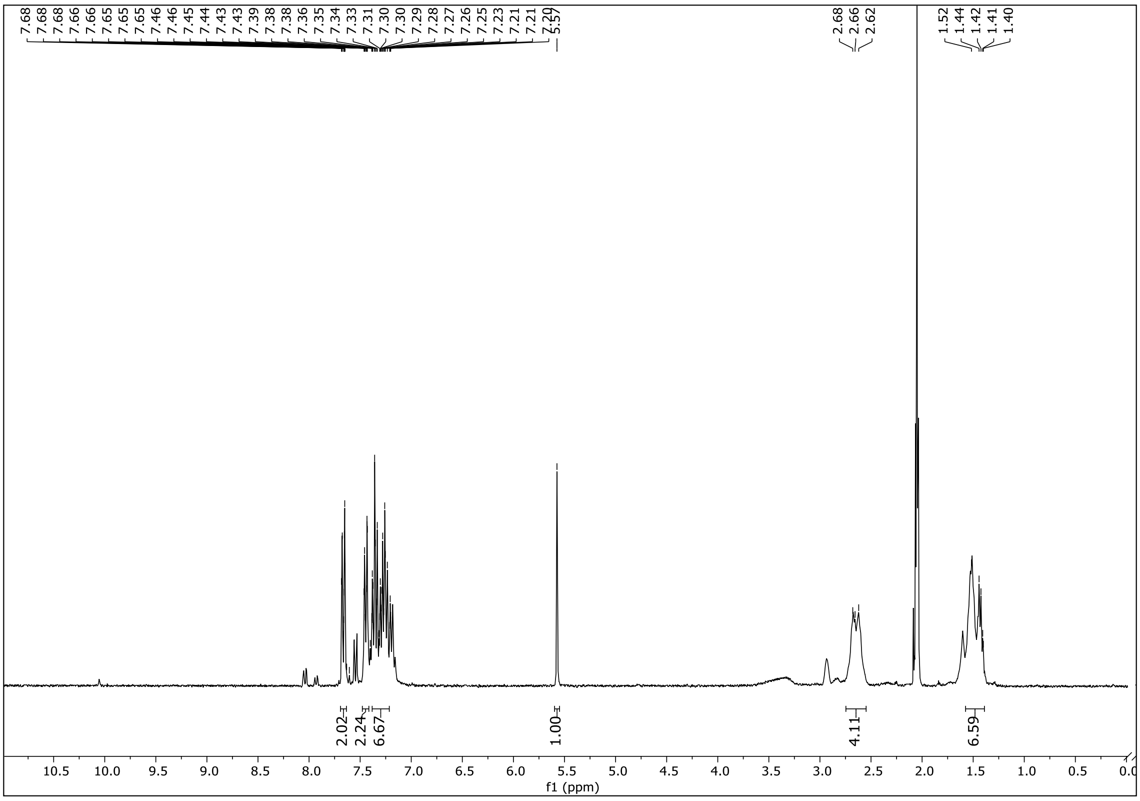

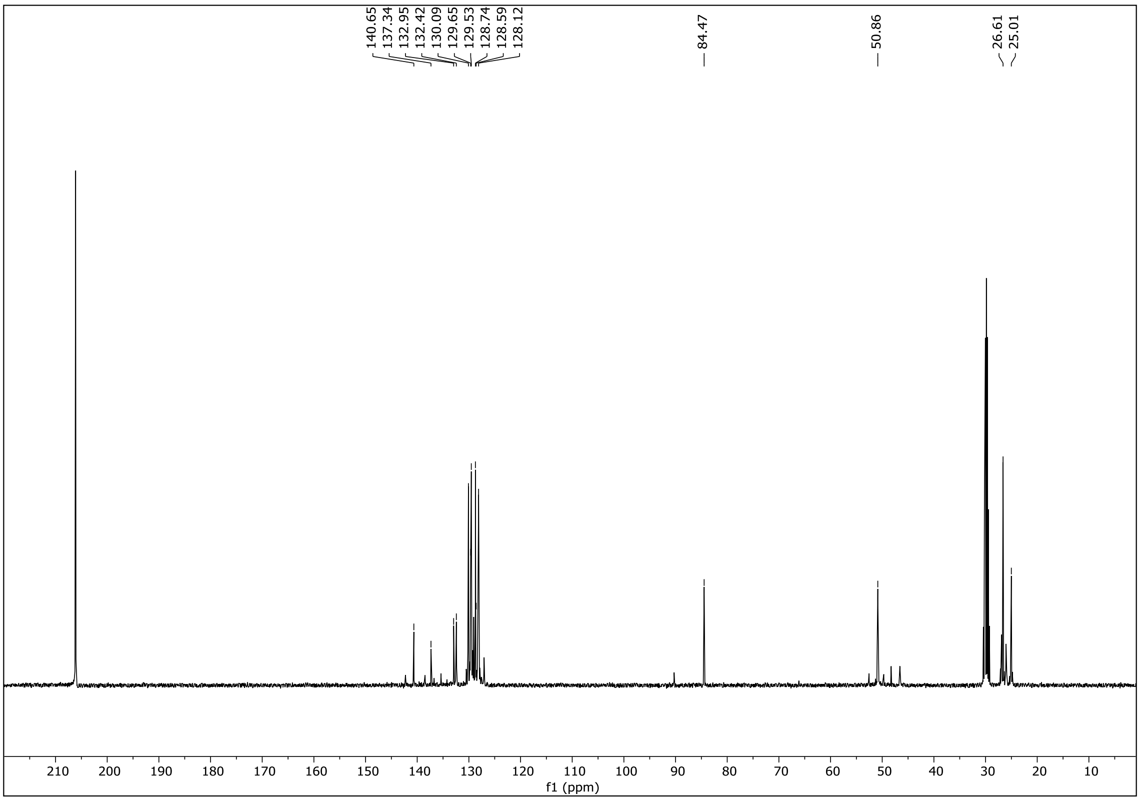

Figure S7. ^{1}H-NMR and ^{13}C-NMR spectra of **6.** The ^{1}H and ^{13}C NMR spectra presented have a mixture of thioaminal **6** and products from hydrolysis, resulting from *in situ* degradation in the NMR tube.

*1-methyl-4-(phenyl(phenylthio)methyl)piperazine (**7**)*

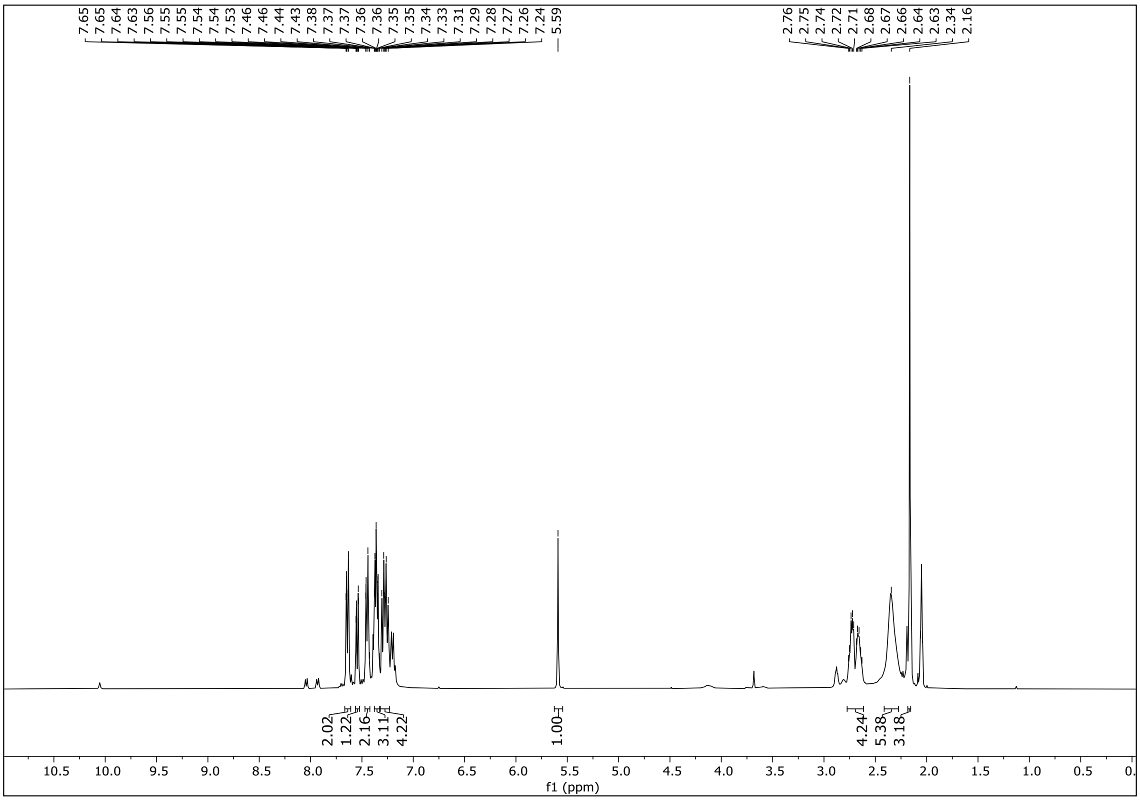

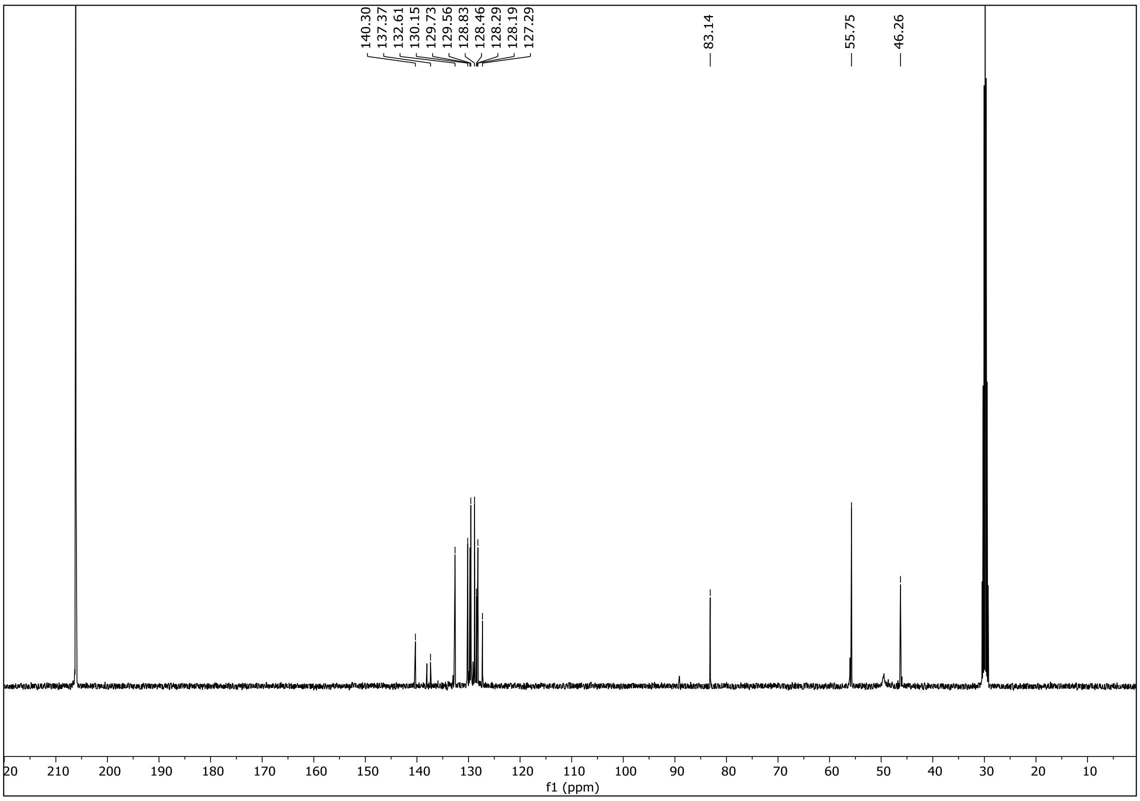

Figure S8. ^{1}H-NMR and ^{13}C-NMR spectra of **7**. The ^{1}H and ^{13}C NMR spectra presented have a mixture of thioaminal **7** and products from hydrolysis, resulting from *in situ* degradation in the NMR tube.

*N,N-dibenzyl-1-phenyl-1-(phenylthio)methanamine (***8***)*

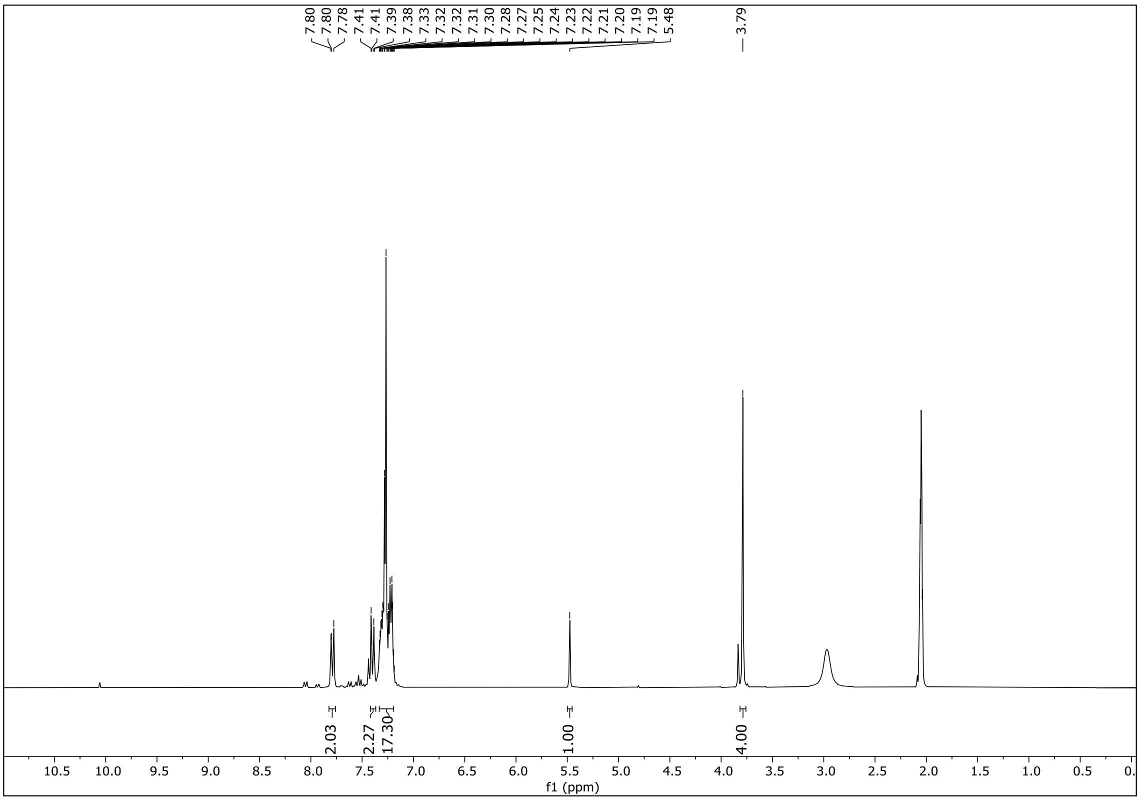

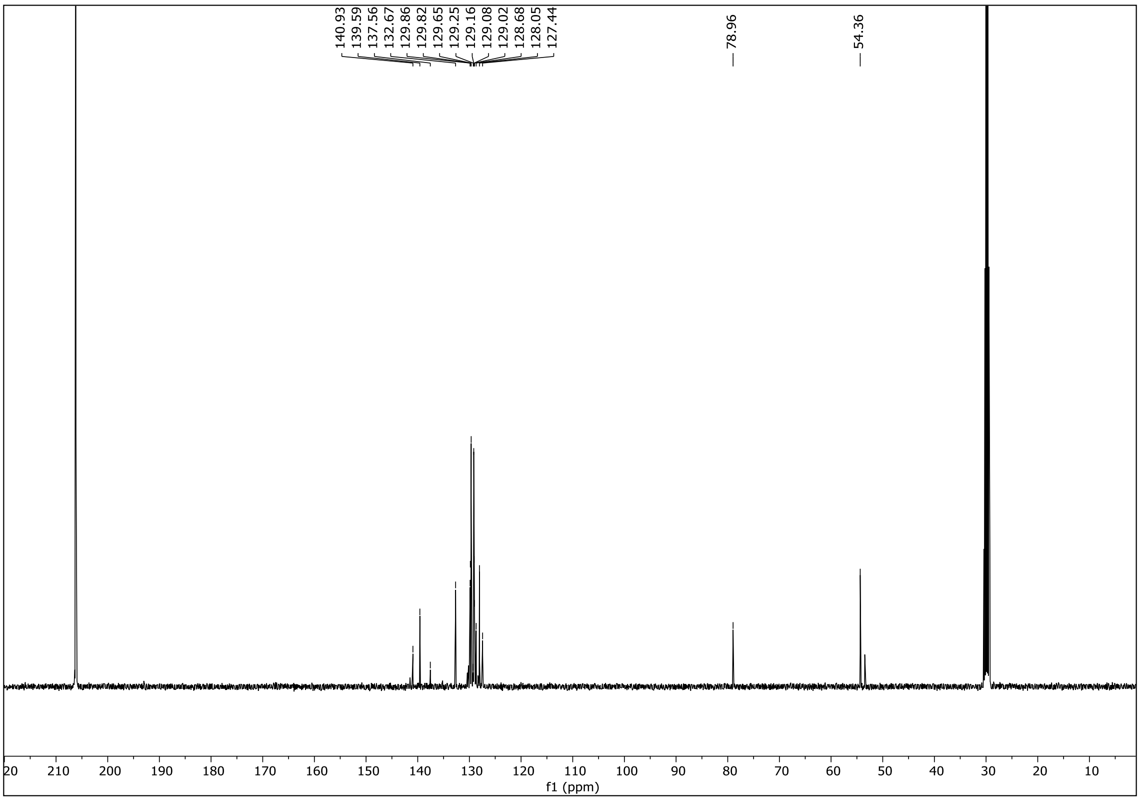

Figure S9. ^{1}H-NMR and ^{13}C-NMR spectra of **8**. The ^{1}H and ^{13}C NMR spectra presented have a mixture of thioaminal **8** and products from hydrolysis, resulting from *in situ* degradation in the NMR tube.

*4 -(phenyl(p-tolylthio)methyl)morpholine (**9**)*

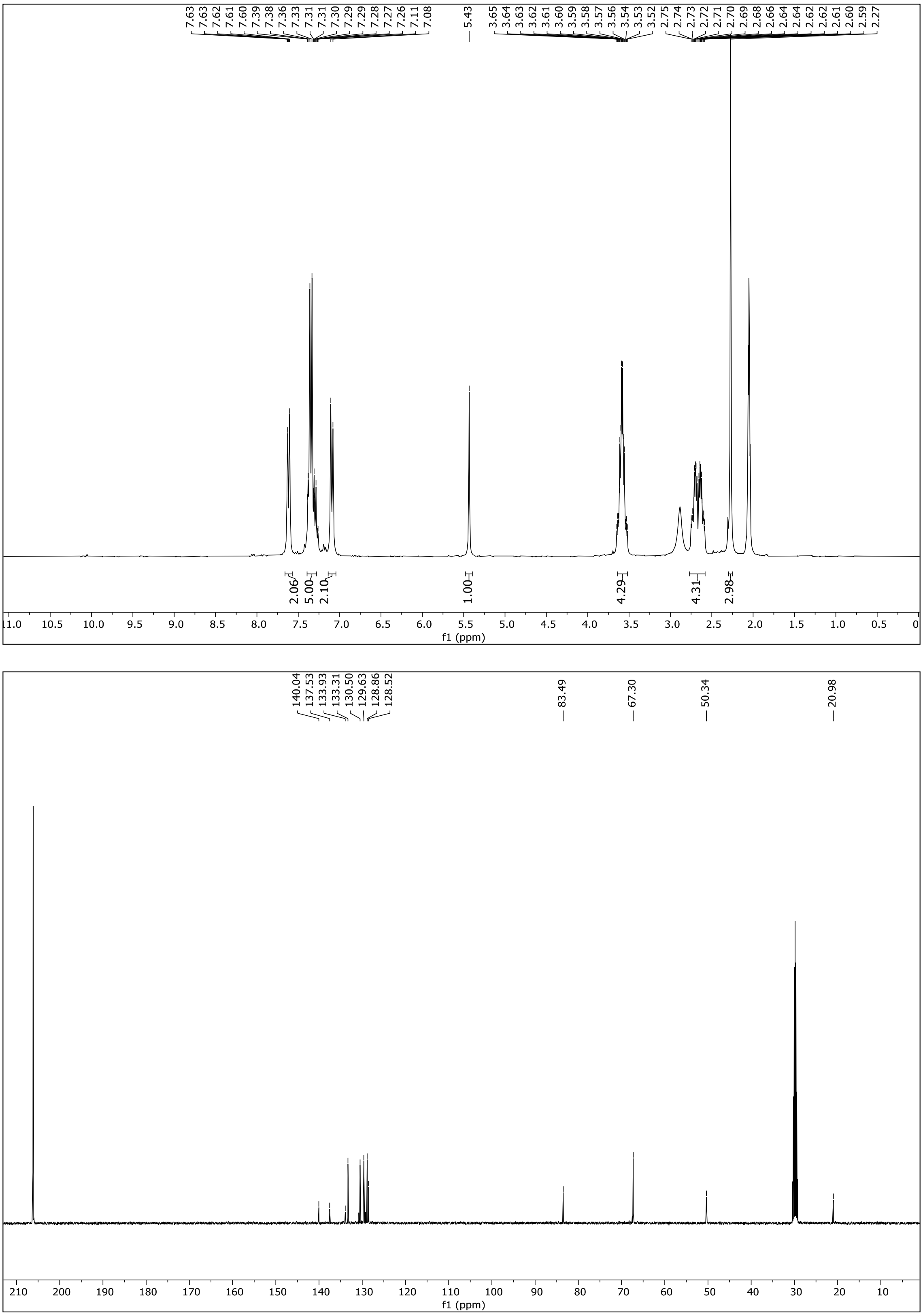

Figure S10. The ^{1}H and ^{13}C NMR spectra of **9**.

*4-(((3,5-dimethylphenyl)thio)(phenyl)methyl)morpholine (**10**)*

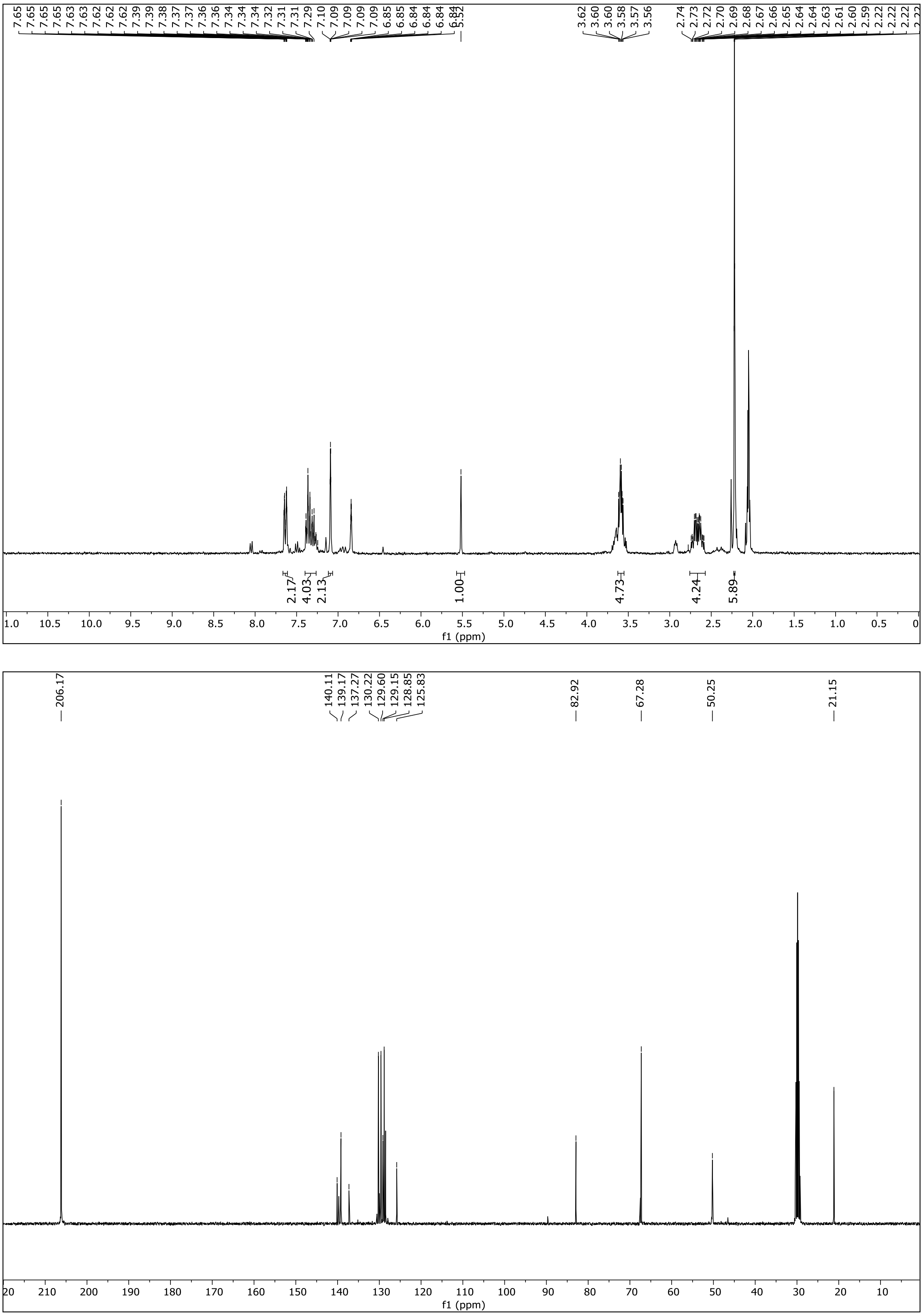

Figure S11. The ^{1}H and ^{13}C NMR spectra of **10.**

*4-(((4-methoxyphenyl)thio)(phenyl)methyl)morpholine (**11**)*

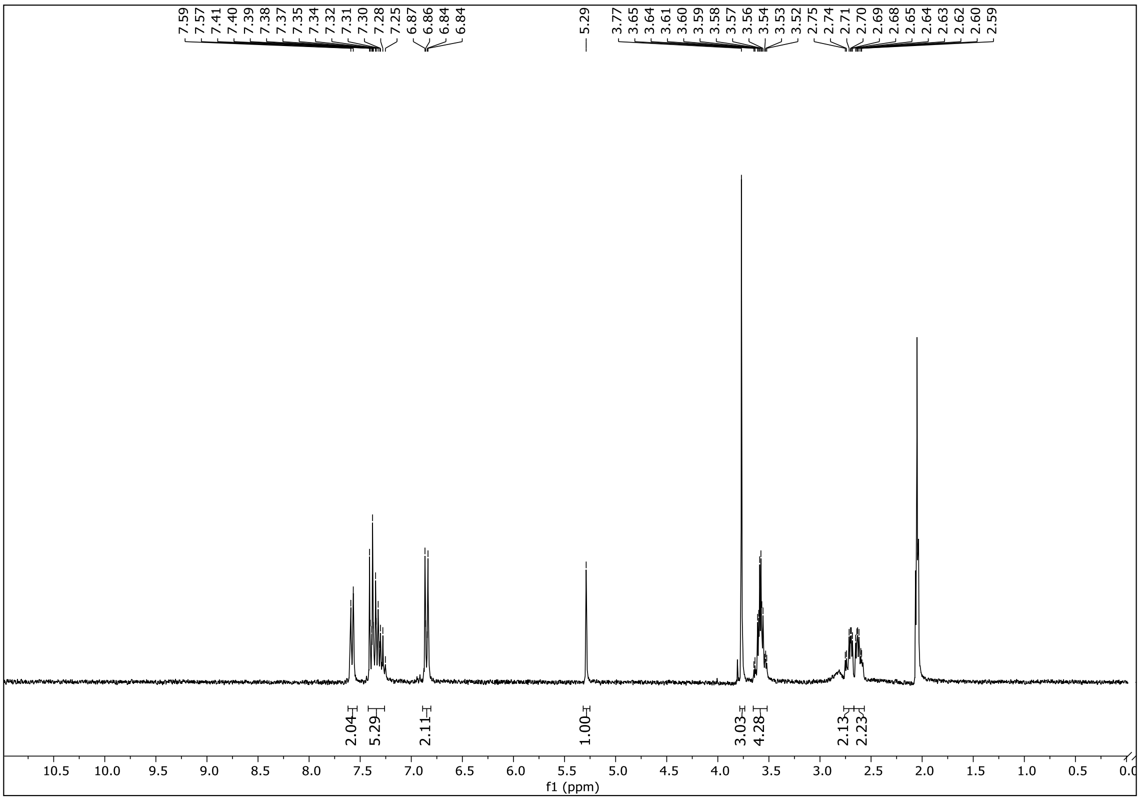

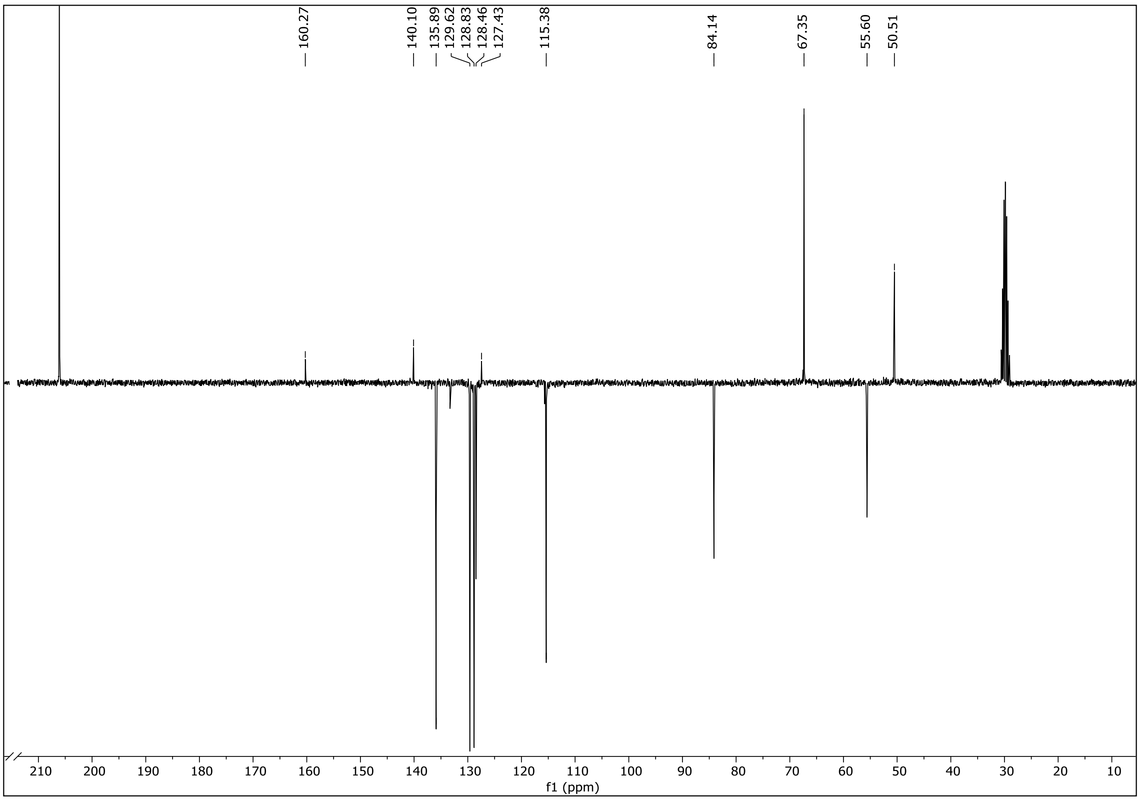

Figure S12. The ^{1}H and ^{13}C NMR spectra of **11.**

*4-(((3,4-dimethoxyphenyl)thio)(phenyl)methyl)morpholine (**12**)*

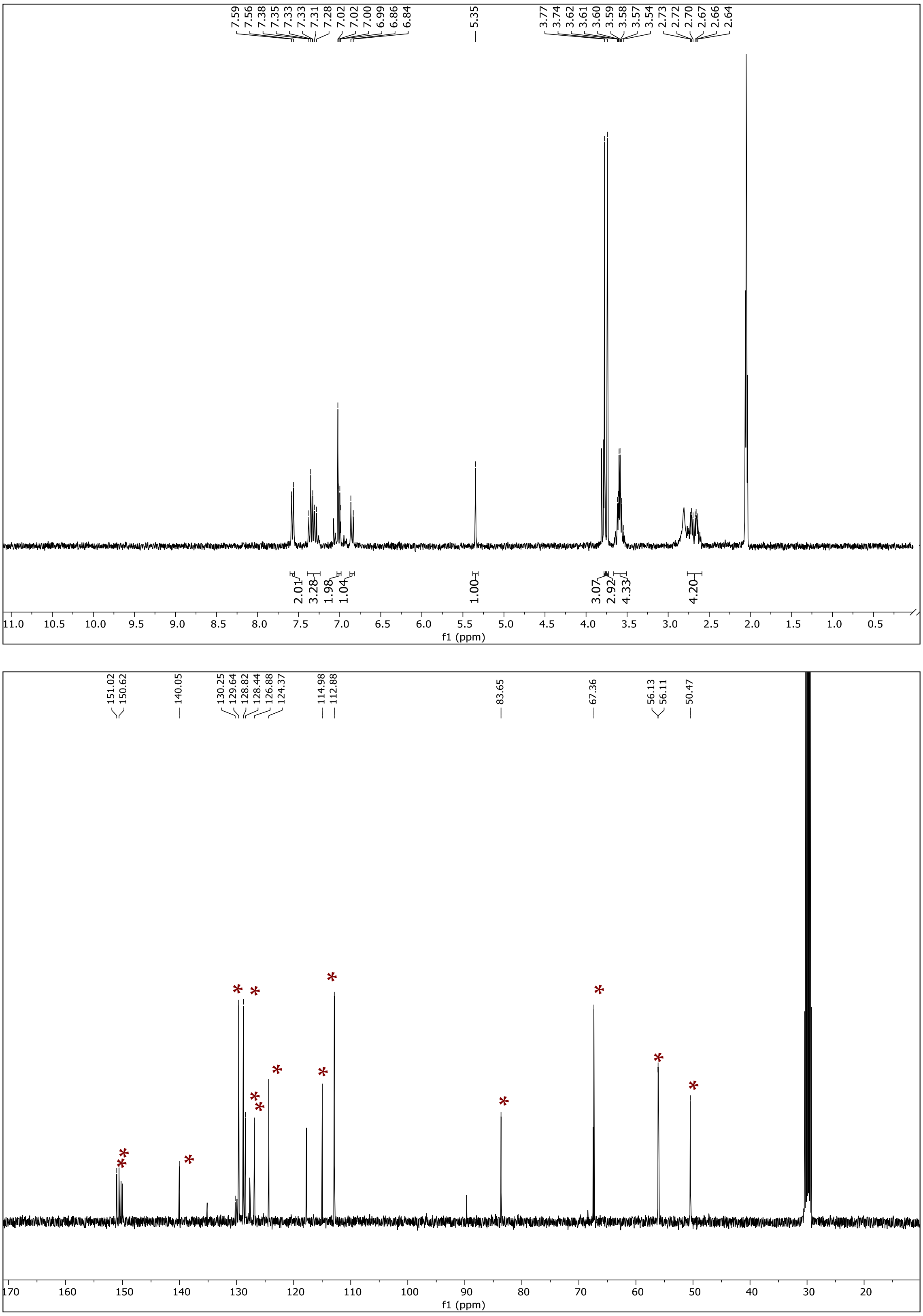

Figure S13. The ^{1}H and ^{13}C NMR spectra of **12.** The ^{13}C NMR spectrum presented has a mixture of thioaminal **12** and products from hydrolysis. Signals from thioaminal **9** are highlighted in the spectrum.

*4-(((4-fluorophenyl)thio)(phenyl)methyl)morpholine (**13**)*

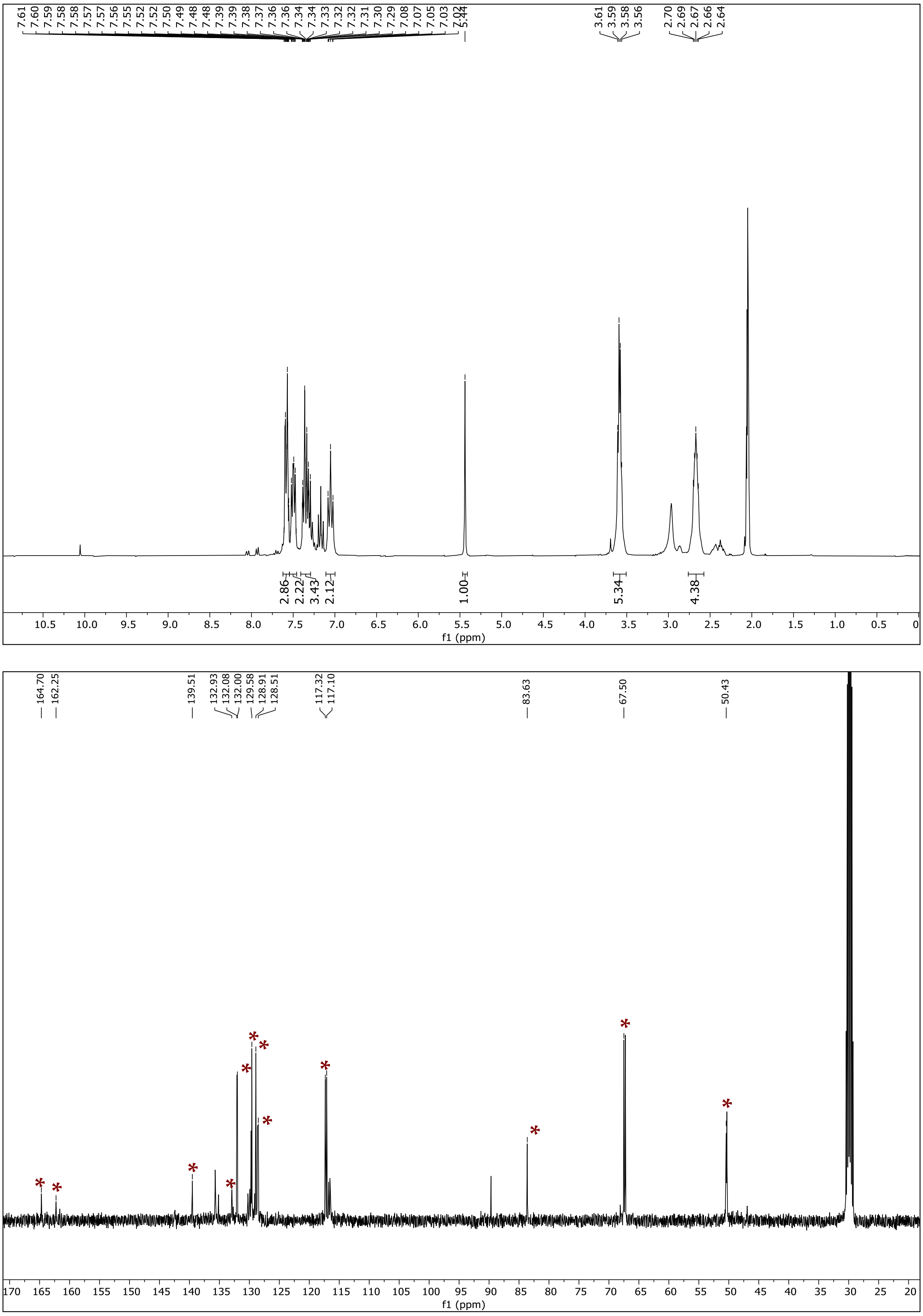

Figure S14. The ^{1}H and ^{13}C NMR spectra of **13.** The ^{13}C NMR spectrum presented has a mixture of thioaminal **13** and products from hydrolysis. Signals from thioaminal **13** are highlighted in the spectrum.

*4-(((4-chlorophenyl)thio)(phenyl)methyl)morpholine (**14**)*

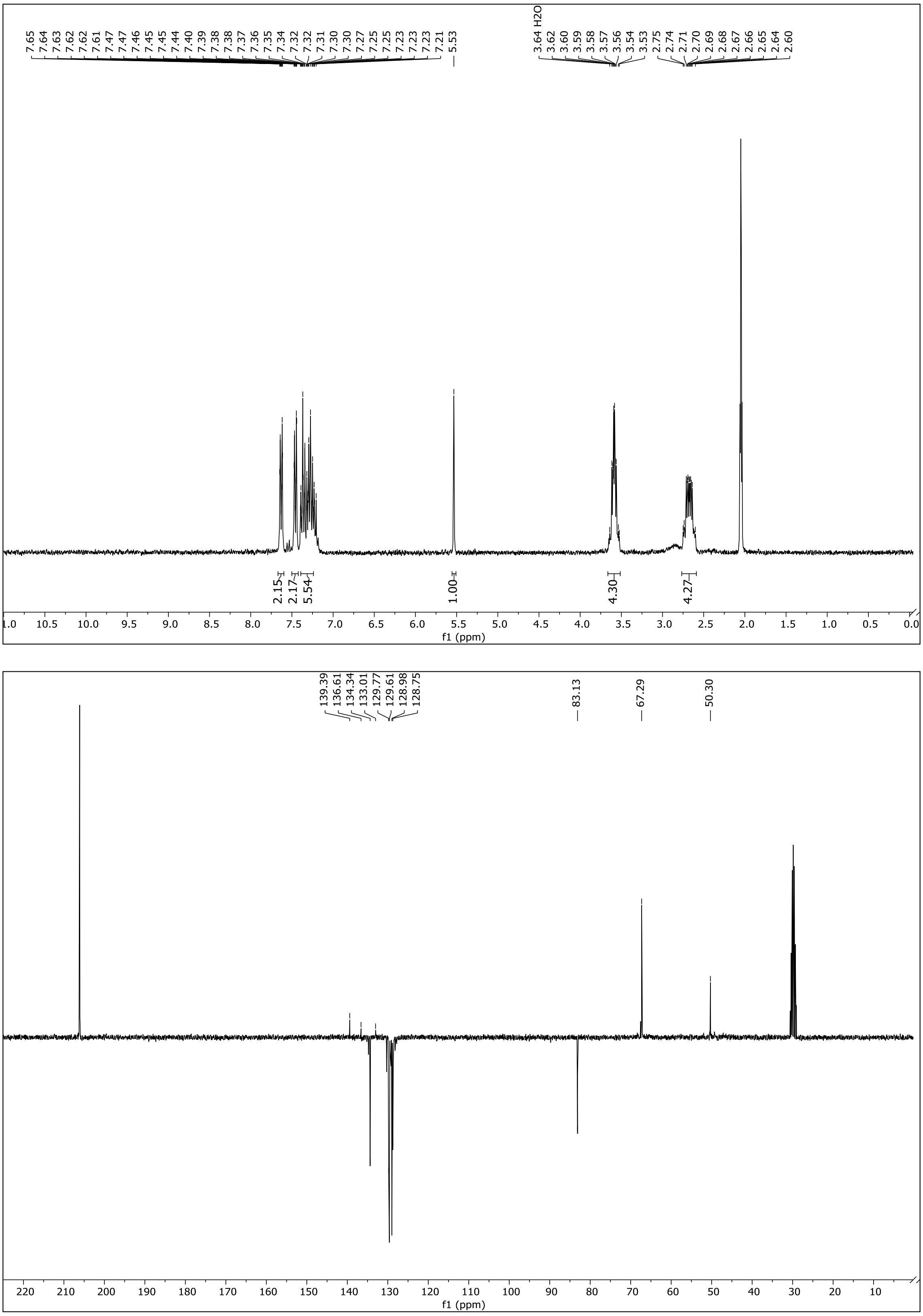

Figure S15. The ^{1}H and ^{13}C NMR spectra of **14.**

*4-(((3,5-dichlorophenyl)thio)(phenyl)methyl)morpholine (**15**)*

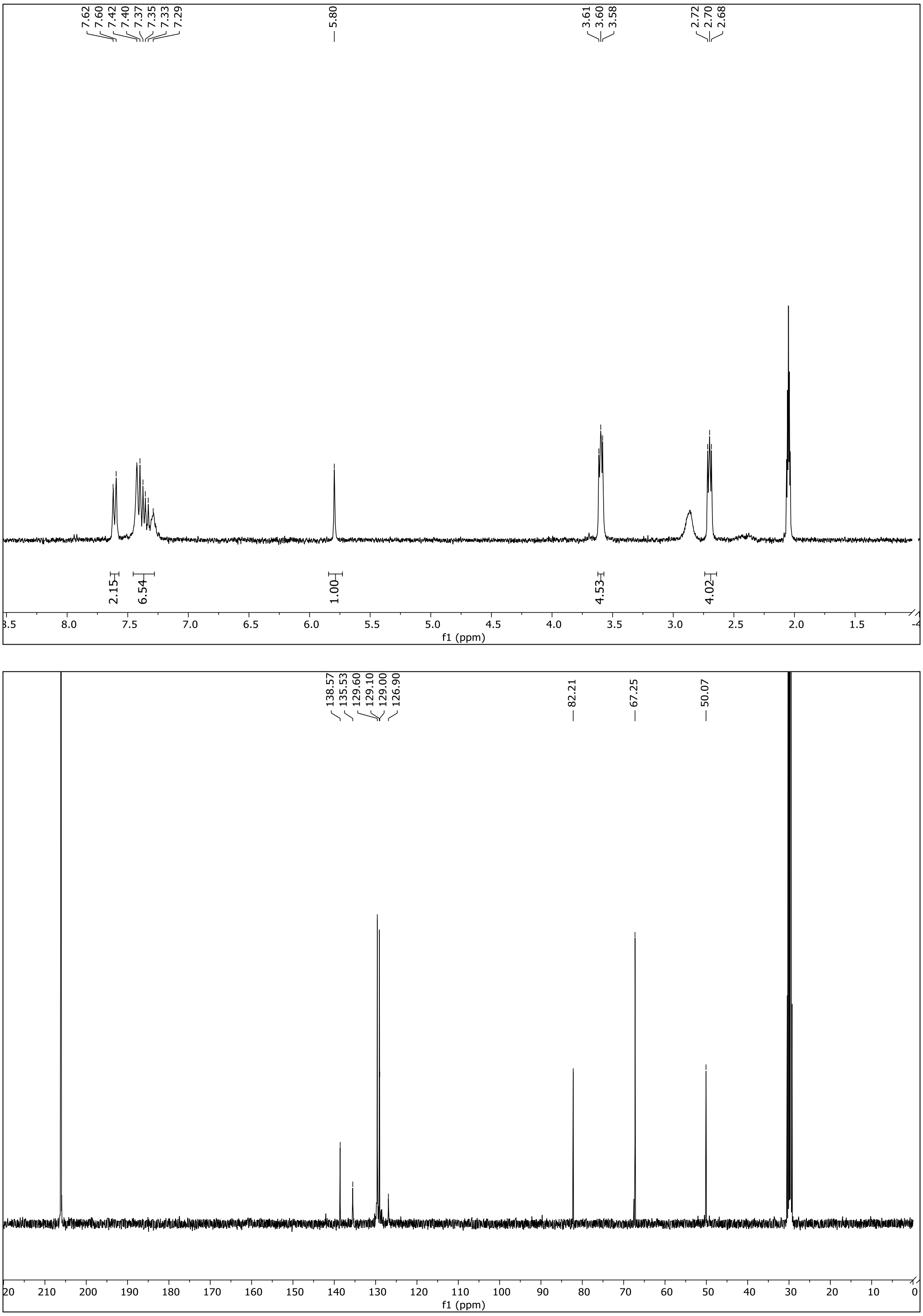

Figure S16. The ^{1}H and ^{13}C NMR spectra of **15.**

*4-((benzylthio)(phenyl)methyl)morpholine (**16**)*

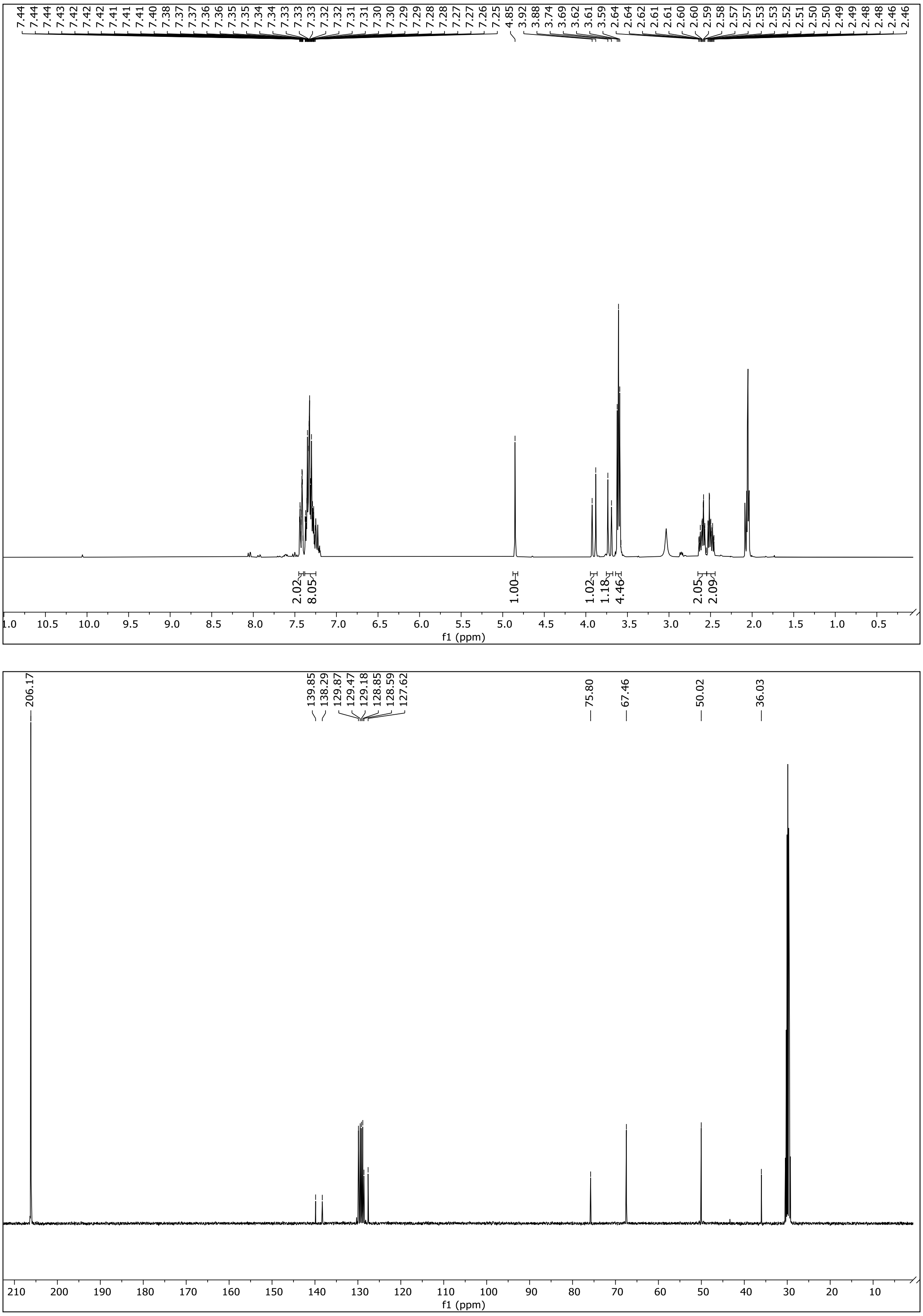

Figure S17. The ^{1}H and ^{13}C NMR spectra of **16.**

4-((phenethylthio)(phenyl)methyl)morpholine (**17**)

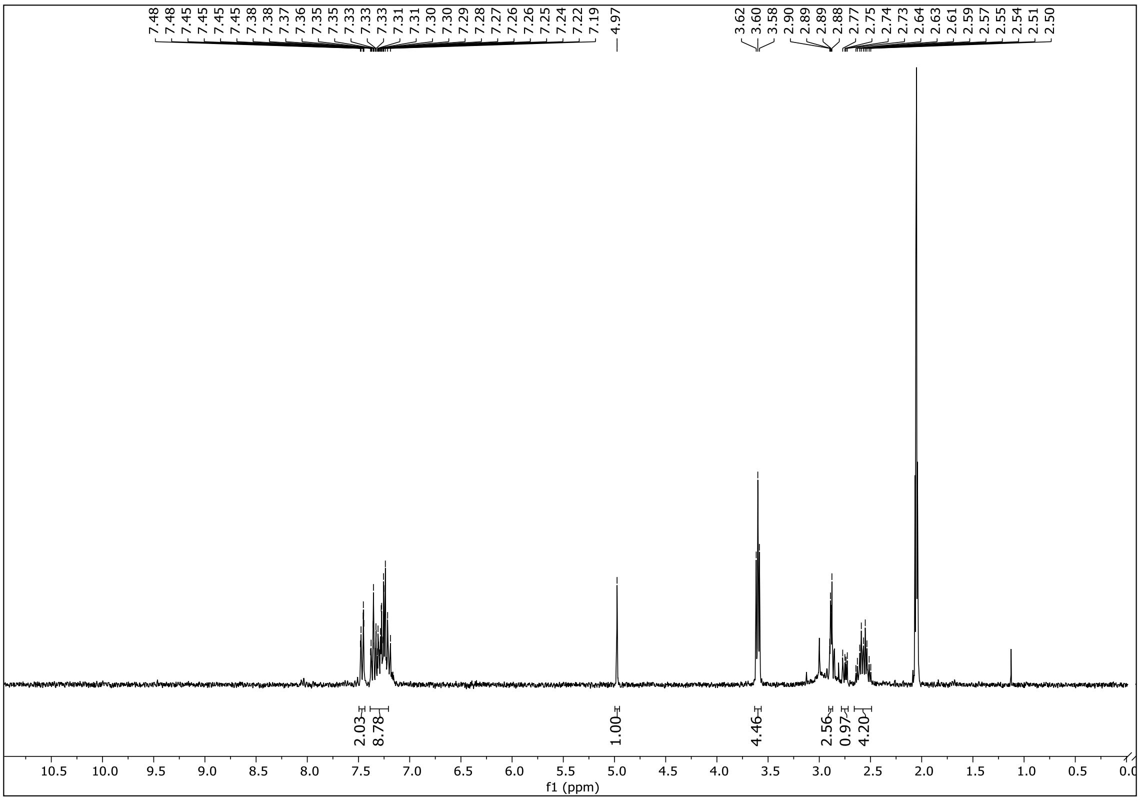

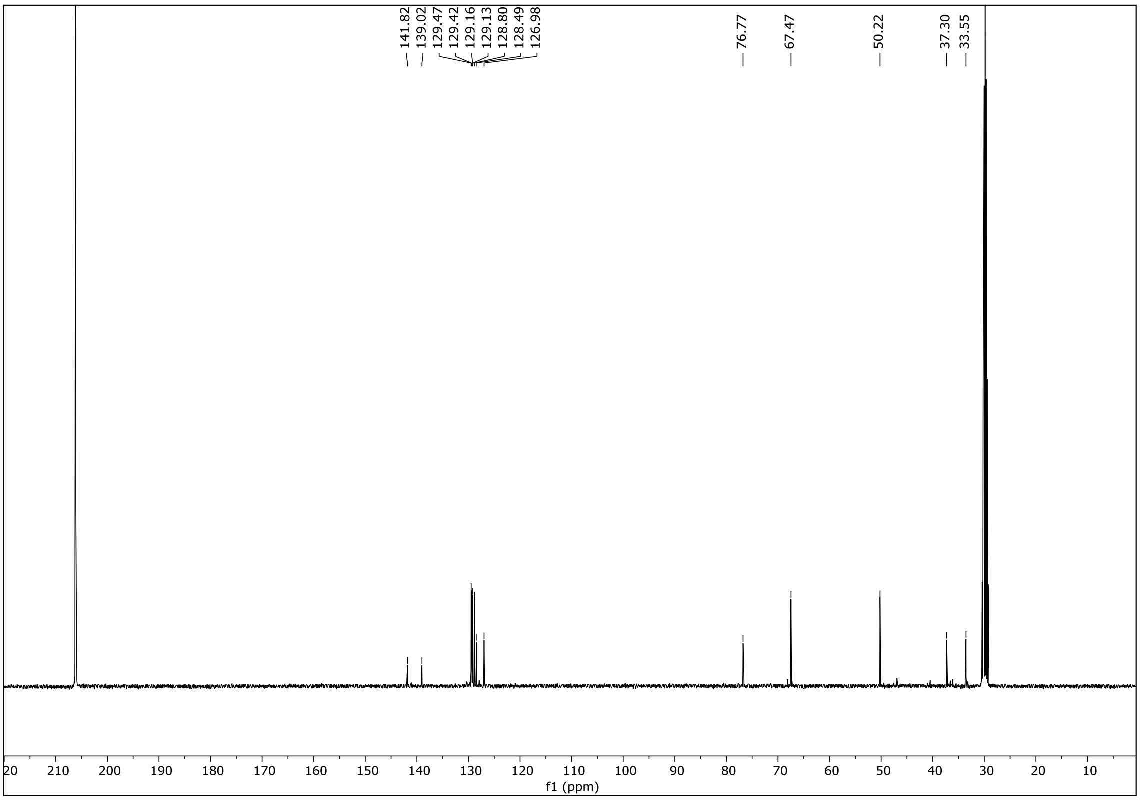

Figure S18. The ^{1}H and ^{13}C NMR spectra of **17.**

*4-(phenyl(propylthio)methyl)morpholine (**18**)*

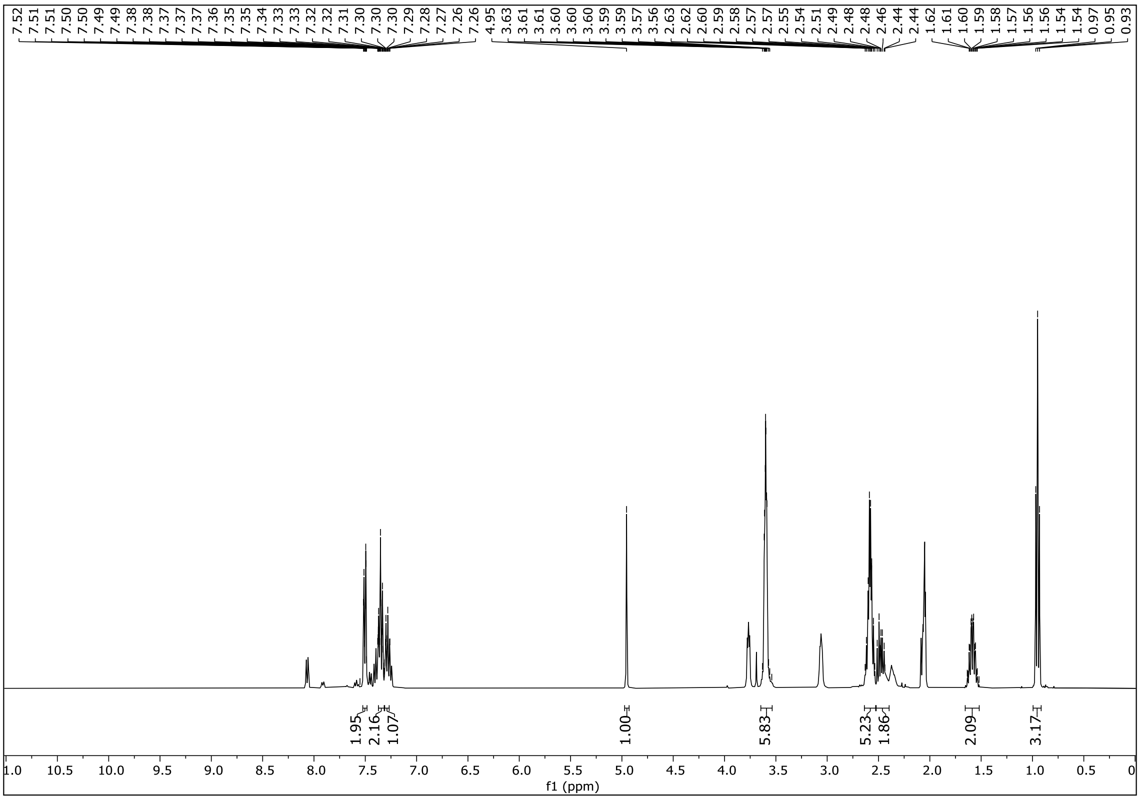

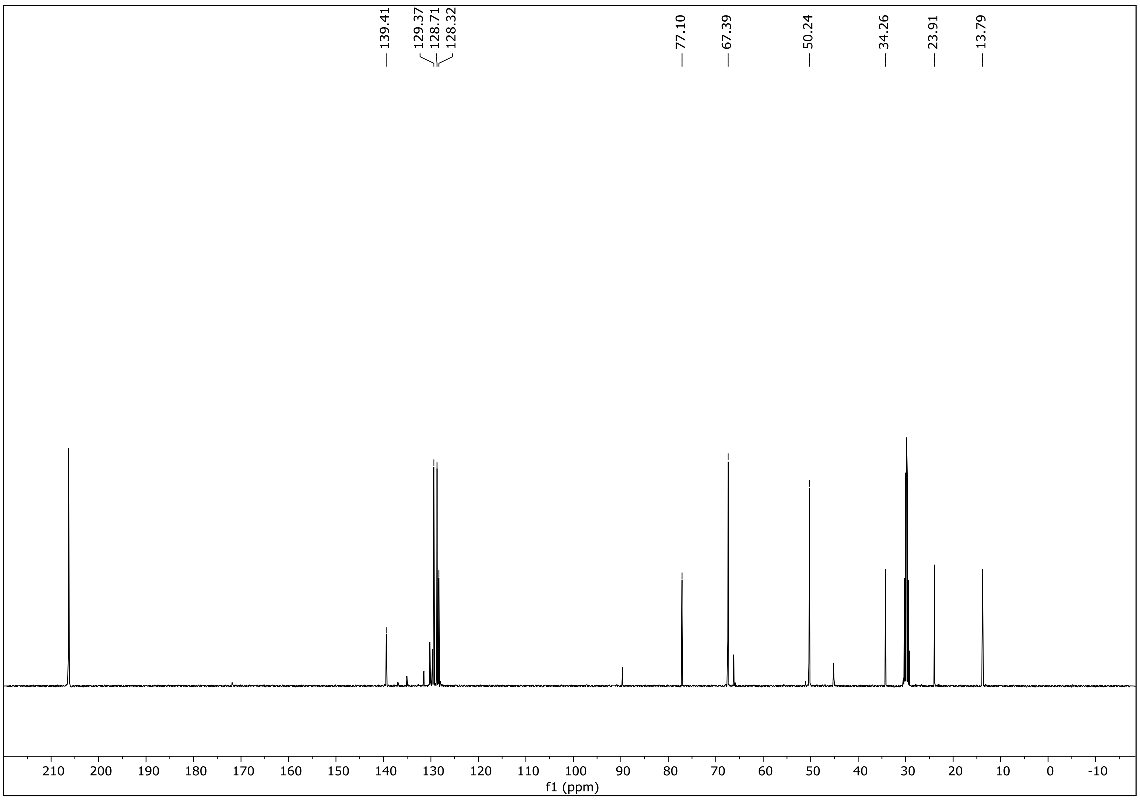

Figure S19. The ^{1}H and ^{13}C NMR spectra of **18.** The ^{1}H and ^{13}C NMR spectra presented have a mixture of thioaminal **18** and products from hydrolysis, resulting from *in situ* degradation in the NMR tube.

*4-((dodecylthio)(phenyl)methyl)morpholine (**19**)*

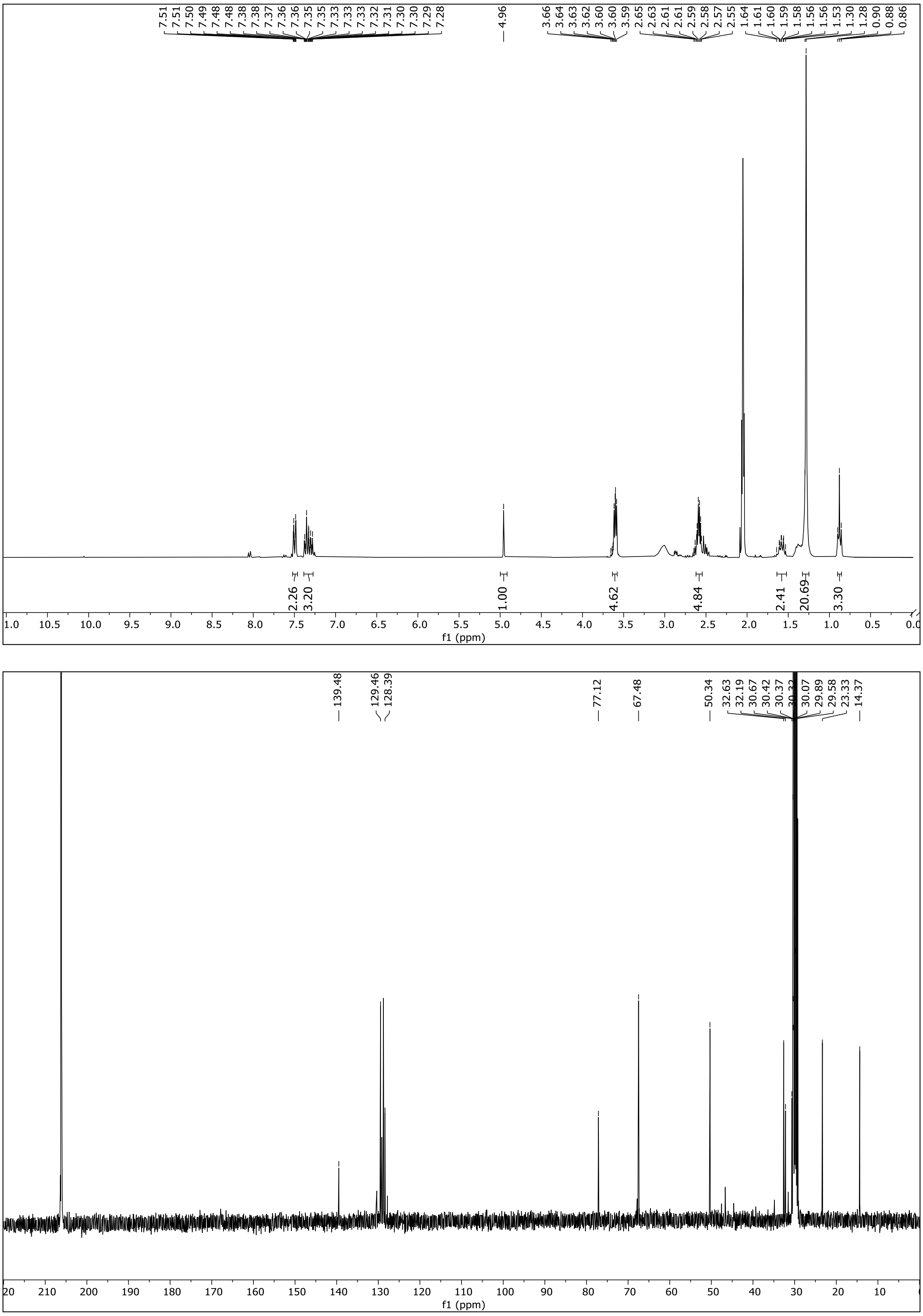

Figure S20. The ^{1}H and ^{13}C NMR spectra of **19.**

UV stability experiments

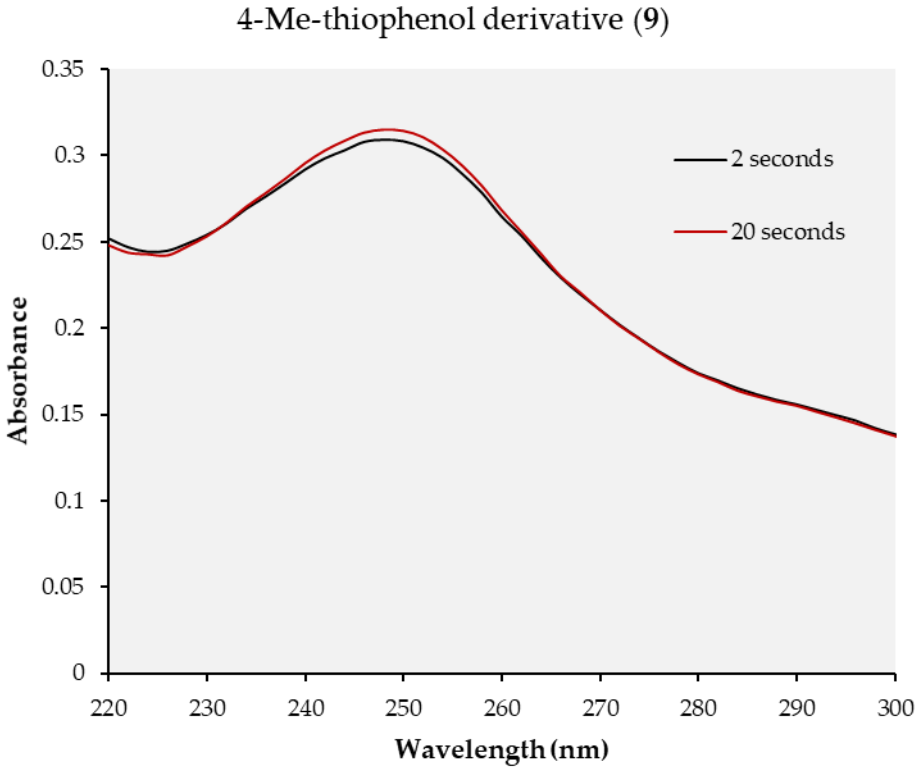

Figure S21. Variation of UV spectrum of thioaminal **9** after 20 seconds at pH 7.

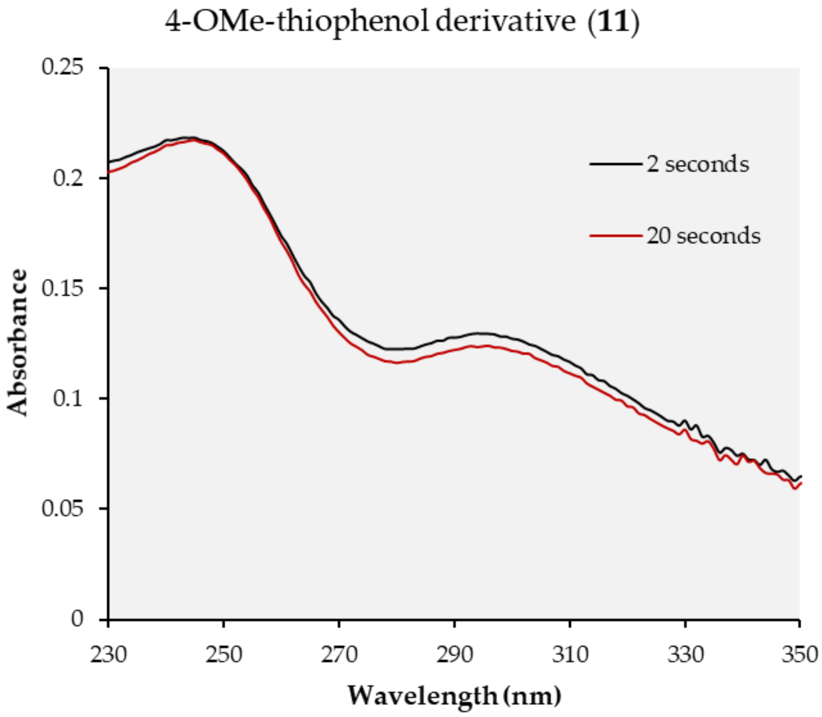

Figure S22. Variation of UV spectrum of thioaminal **11** after 20 seconds at pH 7.

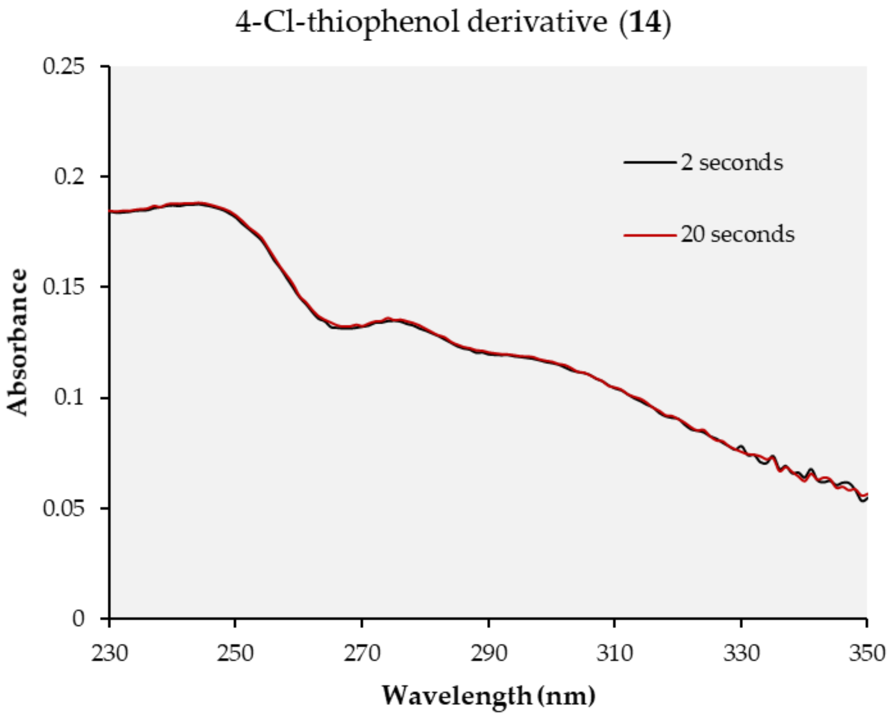

Figure S23. Variation of UV spectrum of thioaminal **14** after 20 seconds at pH 7.

References

1. Fawwaz, M.; Mishiro, K.; Nishii, R.; Sawazaki, I.; Shiba, K.; Kinuya, S.; Ogawa, K. Synthesis and Fundamental Evaluation of Radioiodinated Rociletinib (CO-1686) as a Probe to Lung Cancer with L858R/T790M Mutations of Epidermal Growth Factor Receptor (EGFR). *Mol.* **2020**, *25*, 2914, doi:10.3390/molecules25122914
